# Curated Model Development Using NEUROiD: A Web-Based NEUROmotor Integration and Design Platform

**DOI:** 10.3389/fninf.2019.00056

**Published:** 2019-08-07

**Authors:** Raghu Sesha Iyengar, Madhav Vinodh Pithapuram, Avinash Kumar Singh, Mohan Raghavan

**Affiliations:** Spine Labs, Department of Biomedical Engineering, Indian Institute of Technology, Hyderabad, India

**Keywords:** neuromotor, integrated model, simulator, spinal cord model, design platform

## Abstract

Decades of research on neuromotor circuits and systems has provided valuable information on neuronal control of movement. Computational models of several elements of the neuromotor system have been developed at various scales, from sub-cellular to system. While several small models abound, their structured integration is the key to building larger and more biologically realistic models which can predict the behavior of the system in different scenarios. This effort calls for integration of elements across neuroscience and musculoskeletal biomechanics. There is also a need for development of methods and tools for structured integration that yield larger *in silico* models demonstrating a set of desired system responses. We take a small step in this direction with the NEUROmotor integration and Design (NEUROiD) platform. NEUROiD helps integrate results from motor systems anatomy, physiology, and biomechanics into an integrated neuromotor system model. Simulation and visualization of the model across multiple scales is supported. Standard electrophysiological operations such as slicing, current injection, recording of membrane potential, and local field potential are part of NEUROiD. The platform allows traceability of model parameters to primary literature. We illustrate the power and utility of NEUROiD by building a simple ankle model and its controlling neural circuitry by curating a set of published components. NEUROiD allows researchers to utilize remote high-performance computers for simulation, while controlling the model using a web browser.

## 1. Introduction

Understanding key components of neuromuscular coordination is essential to exploring various aspects of complex locomotion or neuromuscular disorders. Research in neuromotor systems spans across the domains of neuroscience and musculoskeletal systems, and covers the disciplines of anatomy, physiology, and biomechanics. Like any other system, a neuromotor system also consists of components whose scale varies from sub-cellular to systems. Thus, motor systems research in general (Table 1) and computational model development in particular (Table 2) must deal with phenomena across these scales and disciplines. Despite the loss of biophysical realism, several models of various components of the neuromotor systems have been developed as they provide new opportunities to perform *in silico* experiments when *in vivo* or *in vitro* experiments are infeasible. Consequent to proliferation of component models, efforts to build neuromotor systems in part or whole have come into sharp focus. Such system level modeling has mainly advanced in three distinct directions:

**Table 1:** References to published literature in experimental neuromotor systems.

	**Anatomy**	**Physiology**	**Biomechanics**
**Micro**	**Morphological properties like dendritic architecture, projections etc** (Light and Metz, 1978; Schoenen, 1982; Andrew et al., 2003)	**Ion channel mechanisms** (Hounsgaard and Kiehn, 1989; Safronov and Vogel, 1995; Lombardo and Harrington, 2016) **Motoneurons** (Burke, 1968; Fleshman et al., 1981; Hounsgaard and Kiehn, 1989) **NeuroMuscular Junctions** (Land et al., 1981), **Interneurons** (Jankowska, 2001, 2008; Berkowitz, 2008; Jankowska et al., 2009) and **Renshaw Cells** (Renshaw, 1941; Lloyd, 1946; Eccles et al., 1954; Pierrot-Deseilligny and Hultborn, 1979)	**Internal morphology and mechanical properties of muscle fibers and sarcomeres** (Hill, 1953; Bottinelli et al., 1996; Brown et al., 1999; McNulty and Macefield, 2005)
**Meso**	**Histology of spinal laminae, rexed classification** (Rexed, 1954; Sengul et al., 2012)	**Various spinal reflex circuits** (Duysens and Loeb, 1980; Burke et al., 1983; Pierrot-Deseilligny and Burke, 2012) **Central pattern generators** (Duysens and Loeb, 1980; Marder and Bucher, 2001; McCrea, 2001; Marder and Rehm, 2005; Kiehn, 2006, 2016)	**Mechanical properties of muscle like force-length relationships etc** (Durfee and Palmer, 1994; Bottinelli et al., 1996; Maganaris, 2001)
**Macro**	**Spinal cord atlas** (Sengul et al., 2012), **Brain Atlas** (Hawrylycz et al., 2012), **Spinalcord length and gross properties** (Ko et al., 2004), **Stereotactic spinal cord models** (Gabriel and Nashold, 1996; Nadvornik, 2015)	**Control and regulation of locomotion by spinal cord** (Sherrington, 1913; Giulio et al., 2009) **and brain** (Eccles, 1964; Barthélemy et al., 2011) **Local field potentials** (Rall and Shepherd, 1968; Henrik et al., 2011; Reimann et al., 2013)	**Measurement of gross musculoskeletal properties using various techniques motion trackers, ultrasound etc**. (Lee and Piazza, 2009; Sikdar et al., 2014)

*These are categorized based on the scale (micro, meso, macro) and discipline (anatomy, physiology, biomechanics)*.

**Table 2:** References to published literature in computational modeling of neuromotor systems.

	**Anatomy**	**Physiology**	**Biomechanics**
**Micro**	**Morphologically realistic electronic models of motoneuron** (Balbi et al., 2014, 2015) **and Renshaw** (Bui, 2003)	**Computational models of ion channels responsible for unique properties of neurons like PIC, plateau potential** (Booth et al., 1997; Destexhe, 1997; Huss et al., 2008), **Synapses** (Destexhe et al., 1994, 1998; Best et al., 2010), **Neuromuscular junction** (Dionne and Leibowitz, 1982), **Physiologically realistic motoneuron model** (Fuglevand et al., 1993; McIntyre et al., 2002; Cisi and Kohn, 2008), **Interneuron** (Guet-McCreight et al., 2016), **Renshaw** (Bui, 2003; Cisi and Kohn, 2008)	**Models of muscle behavior** (Fuglevand et al., 1993; Brown et al., 1999), **Mechanical models of Muscle Spindle** (Mileusnic, 2006), **and GTO model** (Mileusnic and Loeb, 2006)
**Meso**	**3D localization of neurons** (Gleeson et al., 2007)	**Computational models of CPG** (Matsuoka, 1987; Prinz et al., 2003; Iwasaki and Zheng, 2006; Rybak et al., 2006; Rubin et al., 2009; Shevtsova and Rybak, 2016; Danner et al., 2017)	**Muscle Force-Velocity models** (Cheng et al., 2000), **and joint coordination** (Morasso and Mussa Ivaldi, 1982; Flash and Hogan, 1985)
**Macro**	**Models of spinal connectomes** (Borisyuk et al., 2011)	**Electrical stimulation and neuromodulation of gait** (Capogrosso et al., 2013; Courtine et al., 2016), **Efficacy of stimulation in pain modulation** (Arle et al., 2014a,b), **LFP** (Diwakar et al., 2011), **Optimum electrode geometry for stimulation** (Holsheimer and Wesselink, 1997)	**Models of human locomotion and control** (Geyer and Herr, 2010; Schultz and Mombaur, 2010; Song and Geyer, 2015), **Force estimation during movement** (Seth and Pandy, 2007)

*These are categorized based on the scale (micro, meso, macro) and discipline (anatomy, physiology, biomechanics)*.

### 1.1. Neural Control of Locomotion

One of the important functions of the nervous system is sensorimotor control. Understanding the complex interplay between neural circuits and their control of musculoskeletal elements have been a strong motivation for research in this direction. For example, models of motor unit recruitment (Cisi and Kohn, 2008; Elias et al., 2012; Capogrosso et al., 2013; Heidlauf et al., 2013; Castronovo et al., 2015), computational models exploring central pattern generators (Matsuoka, 1987; Iwasaki and Zheng, 2006; Rybak et al., 2006; Rubin et al., 2009), locomotion and posture control (Jo and Massaquoi, 2004; Shevtsova and Rybak, 2016; Danner et al., 2017) have been developed. Physics based models that mimic locomotion without detailed neural circuitry are also explored (Geyer and Herr, 2010; Song and Geyer, 2015).

### 1.2. Stimulation Therapy

Understanding the effect of electrical stimulation therapies on locomotion has been a key focus area for research, especially due to its downstream clinical applications. This involves understanding of electromagnetic fields generated, the effect on locomotor circuitry and the activation of limbs due to stimulation. Computational models are built to understand the effect of epidural electrical stimulation (EES) on gait (Capogrosso et al., 2013) and modulation of spinal circuits for correction of gait (Courtine et al., 2016). Models are built to study the optimal EES electrode geometry (Holsheimer and Wesselink, 1997) and position (Rattay et al., 2000) for spinal cord stimulation. Modeling of spinal cord circuitry to study the effect of dorsal column stimulation for treatment of neuropathic pain (Arle et al., 2014a) and the effect of scarring (Arle et al., 2014b) have been carried out, which have clinical significance in pain management.

### 1.3. Biomimetics and Robotics

Creating artificial systems with functional equivalence to human movement and locomotion has been an area of interest for technologists. Often, these neurorobotic systems (Krichmar, 2018) do not recreate all the internal mechanisms of a biological system, but instead try to mimic them at a functional level. Such models are explored for tasks such as control of robotic arms (Bakkum et al., 2007; Casellato et al., 2012), navigation and planning (Cuperlier et al., 2007). Platforms which provide an environment to test the behavior of neuromotor models have also been developed (Goodman et al., 2007; Cofer et al., 2010; Falotico et al., 2017). Of late, evolutionary algorithms (Nolfi and Floreano, 2000; Massera et al., 2007) and reinforcement learning (Sutton et al., 1999; Mnih et al., 2015) techniques are also applied in the field of neurorobotics.

The Brain Simulation Platform^1^ is designed for collaborative brain research and visualization (Abdellah et al., 2018).

A significant trend in the last decade has been the emergence of platforms to leverage numerous simulators (Hines and Carnevale, 1997; Gleeson et al., 2007; Ray et al., 2008; Plesser et al., 2013; Bower et al., 2014), model databases (Hines et al., 2004; Halavi et al., 2008), atlases (Lein et al., 2007; Sengul et al., 2012), and programmer-friendly interfaces (Davison et al., 2008; Eppler et al., 2008) to create larger systems (Delorme and Thorpe, 2003; Goodman and Brette, 2008; Sousa and Aguiar, 2014; Cope et al., 2017).

In the context of neuromotor platforms, there have been only a handful of such efforts (Cisi and Kohn, 2008; Kim and Kim, 2018). Development of neuromotor systems and platforms present some unique challenges. A primary challenge is to build systems that straddle across the domains of neuroscience and musculoskeletal biomechanics. Further, to model stimulation therapies, it becomes imperative to fuse models of neuroanatomy and physiology. This multidisciplinary requirement presents a challenge that has not been addressed satisfactorily so far.

Further, creation of a multiscale system involves systematic integration of multiple components at lower scale resulting in a larger component that demonstrates an emergent phenomenon. A controlled integration process must be able to control the choice of components, tune their parameters and arrive at an integrated model. The system response of the integrated model may include the unison of all the component responses and some emergent responses. Current platforms do not support this controlled integration process. This is a limitation that needs to be overcome.

We introduce our platform, NEUROiD, which tries to address multidisciplinary, multiscale simulation and integration. NEUROiD is intended to be a platform for designing neuromotor systems by integration of existing models and information from literature—both neuroscience and biomechanics. NEUROiD currently focuses on the spinal cord and musculoskeletal systems. However, it can be extended to incorporate other supra spinal components involved in motor actions or interface with external models of the supra spinal components. NEUROiD also tries to exploit the stereotypical nature of spinal cord circuits along the rostro-caudal axis. A suite of tools in the platform will enable creation of integrated models. The interface of NEUROiD is designed to encourage non-programmers to actively participate in the design and development of neuromotor system models. NEUROiD is designed as a web-based platform, where the resource intensive simulations are run on a powerful server. The user can interact with the server using a relatively resource constrained computer. The platform is focused on enabling the study of neural control of movement, movement disorders (with locus in spinal cord or downstream) and electrical stimulation-based therapies.

In this paper, we present the first version of NEUROiD and describe the features enabling curation, creation, simulation, validation, and visualization of neuromotor models. We demonstrate the same using a model of the human ankle joint with its controlling neural circuitry in L4 and L5 segments of the spinal cord. We also demonstrate the process of creating an integrated cell model. The integrated model demonstrates cellular level properties such as *spike rate adaptation, frequency-current curve*, and *bistable firing*. and system level responses such as *reflex recruitment curve, orderly recruitment* of the constituent models. Hence, NEUROiD allows creation of models capable of demonstrating a wide array of results spread across multiple scales (sub-cellular to network) and disciplines (anatomy, physiology, biomechanics).

## 2. Methods

### 2.1. Design Philosophy

In order to integrate and build larger system models, an implicit need was to reuse existing models and encourage non-programmers to define, simulate, and visualize neuromotor systems. Thus, the following design principles have guided the development of NEUROiD:

1. Enable model definitions with little or no need for programming. Allow integration of multiple existing models or components.
2. Model neuromotor systems as a multiscale and multidisciplinary systems, where the scales span from sub-cellular to systems, and disciplines span over neuroanatomy, physiology, and musculoskeletal mechanics.
3. Build virtual electrophysiological tools to probe the models and design intervention.
4. Enable compact model definitions by exploiting recurring patterns characteristic to neuromotor systems.
5. Separate compute and memory intensive from lightweight modules, allowing for a client-server architecture.

### 2.2. Architecture

**Figure 1:**
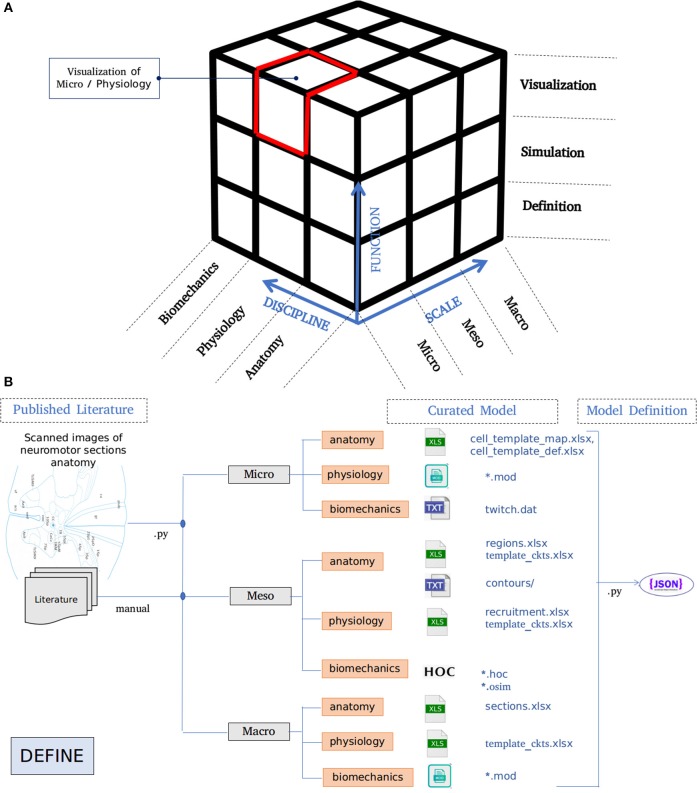
**(A)** Architecture of NEUROiD is best represented as a 3x3x3 cube with function, scale, and discipline being the three axes. The function axis represents major operations performed on a neuromotor system, namely model definition, simulation/virtual experimentation (on the defined model), and visualization (of model and results). The scale axis represents micro, meso, and macro scales of a neuromotor model. The discipline axis represents different disciplines that come together in a virtual neuromotor system, namely anatomy, physiology, and biomechanics. The cubelet corresponding to visualization of micro physiology is highlighted in red. **(B)** Workflow for model curation and definition (read from left to right). Published literature is scoured manually for the parameters required for the model of interest. The parameters pertaining to different scales and disciplines are separated and entered in the relevant tables in xlsx. For instance, micro/anatomy information is curated in cell_template_map.xlsx and cell_template_def.xlsx, Micro/Physiology in relevant .mod files and so on. Python scripts are used to parse the structured curated xlsx sheets to create a composite model definition in the form of a json file.

The architecture of NEUROiD is best represented by a 3x3x3 cube (Figure 1A). The Function axis represents the operations that the user can perform with NEUROiD. The user can define the model, perform a simulation, and visualize the output. Model definition involves creating a neuromotor systems from scratch or using an existing component library provided by NEUROiD. Simulation uses a numerical solver such as NEURON and evaluates the response of model for user defined inputs. Visualization allows inspection and analysis of the model and simulation results. The Discipline axis represents anatomy, physiology, and biomechanics. The Scale axis is used to classify the function and discipline into three hierarchical levels, labeled as micro, meso and macro. At the macro scale, we consider 3D spinal section boundaries, their length, location, and alignment as anatomical properties. Electromyography (EMG) and local field potential (LFP) measurements are examples of Macro level physiology. The angle made at a joint in the neuromuscular model is an example of macro level biomechanical property. At the micro scale, 3D cell morphology, cell sections, position, and orientation are examples of anatomical features. The channel mechanisms inserted in the cell membranes are examples of physiological property. The response of a muscle fiber to a single motor unit action potential forms the micro scale biomechanical property. All properties falling between the macro and micro scales are grouped under meso scale in NEUROiD. For example, the laminae boundaries at various spinal segments and cell groups in laminae form the meso scale anatomical features. Synaptic connections between various cell groups, statistical connection properties such as convergence and divergence ratios form meso scale physiological properties. Changes in muscle fiber length, contraction velocity and afferent feedback form meso scale biomechanical properties. The scales micro, meso and macro loosely translate to *cellular, network*, and *system*, respectively.

### 2.3. Model Curation and Definition

A high-level overview of the operations involved in curation and definition of a model is shown in Figure 1B. This involves study of various relevant *published literature* and extraction of information from them to form a *curated model*. The curated model is a well-structured and concise representation of the model information. NEUROiD mostly uses tables (stored in xlsx files), json^2^ or raw text files to store this information. A *model definition* is created from the curated model. The model definition contains detailed model information obtained by fully enumerating the individual components of model from the curated model. The format of model definition is suitable for generating code used in simulations, rendering 3D model on a web browser and tuning of specific components of the model.

NEUROiD primarily targets simulation of neuromotor systems and the corresponding musculoskeletal systems. Curation of data and creation of model definition represents the *define* plane in the 3x3x3 cube (Figure 1A).

#### 2.3.1. Curation

##### 2.3.1.1. Macro/anatomy

Macro scale (system level) anatomy, forming the Macro/Anatomy component of the model defines sections of spinal cord along the neuraxis. This is known to have clinical significance (Decq, 2003) in localization of the spinal cord. The length of each spinal cord section, the number of sections, their length, 3D location, orientation, alignment, and other such parameters form the macro level anatomical definitions.

The default values used in NEUROiD for the lengths of spinal sections are based on the published results from Ko et al. (2004). We use the “central canal” region in each section of spinal cord, to align the sections.

##### 2.3.1.2. Meso/anatomy

Meso scale anatomy, forming the Meso/Anatomy component of the model is defined by the 3D laminae regions in all the spinal cord sections and cell groups. A functionally homogenous group of cells situated in a 3D lamina region is defined as a “cell group” in NEUROiD (for example, motoneurons innervating the tibialis anterior muscle, present in Lamina 9 of L4 section of spinal cord form a “cell group”). The lamina contours are obtained from scanned high-resolution microscopy images of sections from the spinal cord Atlas (Sengul et al., 2012).

Python scripts are used to extract the contours of laminae in each spinal cord section. The extracted contours for each lamina are stored as lists of points (x and y coordinates). A 3D surface is formed by stitching contours of the same lamina in multiple spinal sections. The 3D space enclosed by this surface is defined as a *region* in NEUROiD. Cells are placed at random locations inside these 3D regions.

For the model and simulations discussed in this paper, cell groups present in the L4 and L5 laminae are identified by literature survey (for example, Watson et al., 2008; Sengul et al., 2012) and tabulated.

##### 2.3.1.3. Micro/anatomy

Micro scale (cellular and sub-cellular level) anatomy forming the Micro/Anatomy component of the model is defined by the cell morphology. Sections in the cell model (for example, soma, axon, and dendrite), their 3D position, number of compartments, diameter, length, and connection between sections form the micro level anatomical definitions. This curated information is stored in a structured, tabulated form.

Neuronal parameters from multiple published literature (Fuglevand et al., 1993; Booth et al., 1997; Destexhe, 1997; Courtine et al., 2016) are used in NEUROiD to create the default library of cell models.

The cell models from this default library are used to create cell instances in various cell groups of the spinal cord model.

##### 2.3.1.4. Macro/physiology

Algorithms and parameters used for evaluation of local field potential (LFP) forms an important part of Macro/Physiology component. Mathematical computation of the local field potentials, which are generated by complex interactions of current sources can be evaluated in NEUROiD. Extension of the same to study ephaptic interactions is planned for a future release. LFP evaluation techniques have been used in single neuron models (Holt and Koch, 1999; Diwakar et al., 2011) and in network models (Henrik et al., 2011; Reimann et al., 2013). LFPSim (Parasuram et al., 2016) is a NEURON-based tool for calculating the local field potentials in various parts of simulated brain and spinal cord. Point source approximation (Rall and Shepherd, 1968; Holt and Koch, 1999), line source approximation (Gold et al., 2006) and low pass RC (Bédard et al., 2004), which are the popular models for analyzing extracellular current sources, can be simulated in LFPSim.

NEUROiD uses LFPSim to evaluate the LFP at any user selectable point in 3-dimensional space of the model. Extracellular potentials are simulated by LFPSim using the *extracellular* mechanism available in NEURON.

##### 2.3.1.5. Meso/physiology

**Figure 2:**
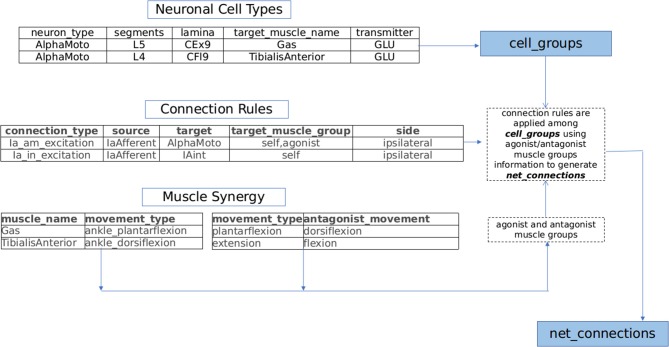
NEUROiD generates net connections between various cell groups using a *muscle synergy based motor circuit generation technique*. Lists of agonist and antagonist muscles are derived from the tabulated set of movement types mediated by each muscle (*muscle synergy*). Repeating connection motifs are defined under *connection rules*. The neuronal cell groups associated with muscles are specified under *neuronal cell* types. NEUROiD's muscle synergy based connection generator connects various cell groups associated with various muscles according to the connection motifs specified.

The Meso/Physiology component of the model comprises of the synaptic properties between and within various cell groups. The combination of source cell group, destination cell group, and synaptic properties for connecting synapse is defined as a “net connection” in NEUROiD. Physiological information related to the synapses (for example, neurotransmitter, connection strength, and delay) are curated from published literature and stored as connection properties in a structured form. The connection rules and statistics of synaptic connections can either be explicitly specified in NEUROiD, or automatically generated. For automatic generation, NEUROiD currently supports two methods; a functional method and another based on anatomy, which are described below:

1. **Muscle Synergy based Sensory Motor Circuit Generation:**
  The musculoskeletal behavior is a mirror of neuronal activity in the brain and spinal cord. This phenomenon is substantiated by the somatotopic organization of central nervous system (Swett and Woolf, 1985; Cohen et al., 1991; Hauk et al., 2004). The fact that spinal cord can produce partial or complete locomotion devoid of supra-spinal inputs signifies the presence of local spinal based neuronal controller (Mushahwar and Horch, 1998; Giszter and Hart, 2013; Desrochers et al., 2019).
  The spinal controllers comprising of sensory, motor and interneurons are organized into intricate circuits catering to stereotypical motor functions (Shefchyk et al., 1990; McCrea and Rybak, 2008; Kiehn et al., 2010; Talpalar et al., 2011; McLean and Dougherty, 2015). The motor circuits responsible for different motor functions are topographically organized in specific regions of the spinal cord (Tresch et al., 2002; Lemay and Grill, 2004; Moritz et al., 2007; Overduin et al., 2014). Such an organization of spinal networks shows the significance of anatomical localization and its functional interpretation.
  Further the connection modalities within the proprioceptive feedback circuits and central pattern generators (CPGs) rely strongly on muscle synergy (Windhorst, 2007; Markin et al., 2012; Takei et al., 2017; Desrochers et al., 2019). Drawing inspiration from spinal circuit organization pattern and based on an extensive review of proprioceptive circuits and spinal interneuronal pathways (Jankowska, 1992, 2001, 2008; Pierrot-Deseilligny and Burke, 2012), we build a framework capable of generating sensory-motor circuits for musculoskeletal functions. This generated circuitry is projected in a 3D segmental map of Spinal cord in NEUROiD. The technique used to generate the circuitry using the curated information is illustrated in Figure 2 and explained below:
  - *Neuronal Cell Types*: Motoneurons, interneurons, and neurons of dorsal root ganglion (DRG) are major constituent neuronal types of the sensory-motor circuitry in spinal cord (Loeb, 2014; Côté et al., 2018). Each of these classes of neurons are associated with either sensory, motor or modulatory aspects of nervous system. In NEUROiD, the anatomical locations (specified as spinal segment and lamina) of these neurons and their properties (for example, neurotransmitter, target muscle, and number of neurons) are tabulated and an exhaustive list of cell groups is obtained.
    For example, the details in neuronal cell types table of Figure 2 results in the creation of alphamotoneuron cell group at designated crural extensor (lamina 9) region of L5 segment in the 3D spinal map of NEUROiD with gastrocnemius as its target muscle. Thus, unique cell groups defined by a combination of spinal segment, spinal cord side, lamina, neuron type, and sub-cell type (target muscle) are formed in NEUROiD. The cell group formed in the above example is called as Human_L4_CEx9_L_AlphaMoto_Gas. Please refer to the Supplementary Material for further information.
  - *Connection Rules*: The net connections among the various cell groups are obtained through connection rules in NEUROiD. The stereotypical connection rules established via histological tracing studies (Matsuyama et al., 2006; Levine et al., 2014) or electrophysiological and neurophysiological characterization (Jankowska, 1992, 2008; Zhang et al., 2010; Pierrot-Deseilligny and Burke, 2012) can serve as a connection template to generate net connections in NEUROiD. Each template is specified by the source neuron type, target neuron type, synaptic properties, and target muscle group.
    For example: IaExcitation pathway defined by “IaAfferent neurons from muscle spindles, upon stimulation can monosynaptically excite alphamotoneurons, targeting both homonymous muscles and agonist muscles groups of ipsilateral side” (Jankowska, 1992), is specified as a connection template in NEUROiD as shown in connection rules table of Figure 2. The source cell group is defined as the IaAfferent cell group and the destination cell group as the alphamotoneuron. In this case, the destination cell group innervates the same muscle group from which the IaAfferents arise. These rules are used to create template net connections between various cell groups. The motoneuron groups to be used for specific connections are derived using the muscle synergy information as described below.
  - *Muscle synergy*: Coordinated activation of a group of muscles producing a particular movement constitutes toward muscle synergy. Each movement can further be decomposed into flexion and extension synergies. The muscles that coordinate to either flex or extend a joint can be grouped as agonists while the muscles of flexion and extension forms an antagonist pair (Fernando, 2017). This information is specified in NEUROiD as shown in muscle synergy tables of Figure 2. The muscles and their movement types along with a list of human movement types and their antagonist movements are tabulated. Using both tables, the muscles are segregated into agonist and antagonist groups.
    Plantar-flexion and dorsi-flexion are defined as antagonistic movement types in a table as seen in Figure 2. Along with this in another table, we define that the gastrocnemius and tibialis anterior muscles are the major contributors for plantar-flexion and dorsi-flexion respectively. This is used to obtain the agonist and antagonist muscle groups to create net connections based on connection rules (here gastrocnemius and tibialis anterior form antagonist group of muscles).
    Based on muscle synergy and connection rules, an exhaustive list of net_connections present in the model is created.
  Further details on this circuit generation technique can be found in Supplementary Material.
2. **Axon-Dendrite and Axon-Soma co-locations based Circuit Generation:**
  The co-location of source cell group axon and destination cell group soma or dendrite are used as indicators of potential synaptic connections. This putative equivalence, commonly referred to as Peters' rule (Peters and Feldman, 1976; Braitenberg and Schüz, 1991; Peters and Payne, 1991) has been quantitatively confirmed (Peters and Feldman, 1976; Binzegger et al., 2004; Krishnaswamy et al., 2015), as well as challenged (Li et al., 1992; Freund and Buzsáki, 1996) in some cases. Recent meta-studies (Rees et al., 2017) however, show that this is a reasonable assumption to make, as most of the exceptions to Peter's rule were attributed to a couple of families of neurons with well-known targeting specificity. When enabled by the user, NEUROiD uses the cell group and its 3D positional information to obtain potential net connections using this rule.
  Either one or both of the methods described above can be used to generate net connections in NEUROiD.

##### 2.3.1.6. Micro/physiology

The Micro/Physiology component of the model is defined by the channel mechanisms inserted into the cell models, and their properties. NEUROiD database of components largely uses .mod files published in existing literature or downloaded from public repositories such as ModelDB (Hines et al., 2004). In future, the micro scale physiology information too will be stored in a structured, tabulated form. This will allow NEUROiD to generate the cell mechanism .mod files using the stored parameters.

The channel mechanisms used in the examples used in this paper are obtained from the published works of Courtine et al. (2016), Booth et al. (1997), Destexhe (1997), and McIntyre et al. (2002). All references to mod files in this paper imply NEURON .mod files.

##### 2.3.1.7. Biomechanics

The generated output of a simulated neuromotor system in NEUROiD is best appreciated when it is interfaced to a musculoskeletal model.

**Figure 3:**
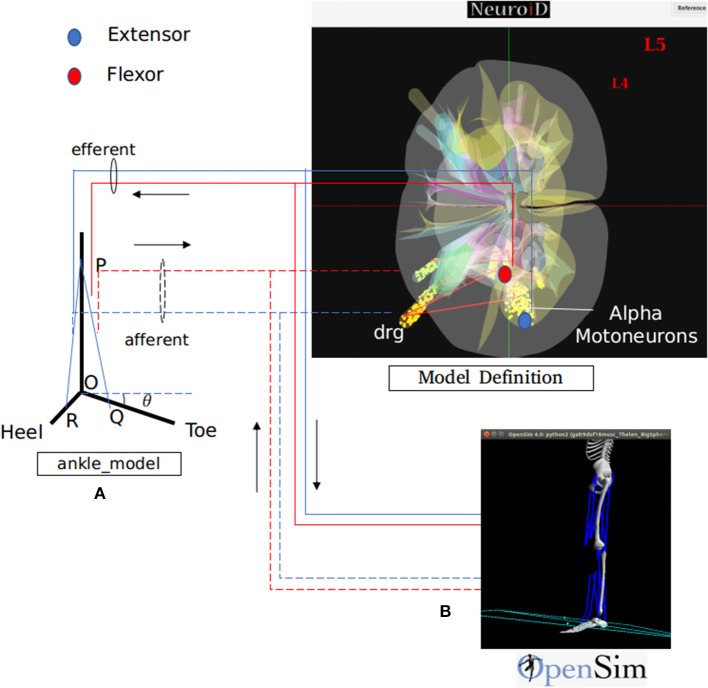
NEUROiD interfaces to biomechanical models for a complete and realistic neuromotor simulation. As an example, we have integrated NEUROiD with two sample biomechanical models of ankle joint for this paper. **(A)** A simplistic rigid body model of the ankle joint. The differential equations governing the model are coded in a mod file. The state of this model is evaluated at every timestep by NEURON along with rest of the neuromotor system model. **(B)** An OpenSim (Seth et al., 2011) model of lower body mechanics (Delp et al., 1990). OpenSim is used to solve the state of this model at every time step and runs as a co-simulation along with NEURON. The solid lines represent the efferent connections and the dashed lines represent the afferent connections. The extensor connections are shown in blue and the flexor connections are shown in red. The activation of motoneurons are used to excite the muscles in one of the two musculoskeletal models. The afferent firing frequency is evaluated by the musculoskeletal model.

We hereby show two different methods for definition, simulation and visualization of the biomechanics, namely (a) through a native mechanism within NEUROiD and (b) through use of the OpenSim software. In the former method, the user is responsible for encoding the differential equation corresponding to all the musculoskeletal segments, their interactions and the like by means of differential equations. The differential equations may be defined in the system by means of NEURON .mod file mechanism (Figure 3A). The second method uses OpenSim To define the musculoskeletal model. NEUROiD provides a glue code for NEUROiD to interact with Opensim and run a co-simulation (Figure 3B).

Motoneuron activations are calculated based on the action potentials observed at their axon terminals. This activation is used to excite the corresponding muscles in the musculoskeletal model. The Lengths, velocities of contraction and the joint angles are returned by the musculoskeletal model, which are then used to calculate the afferent firing.

The Macro/Biomechanics component consists of the joint angles of the musculoskeletal model. The Meso/Biomechanics component includes the transfer function between motoneuron activation and resulting change in muscle length, velocity or force, while the Micro/Biomechanics component consists of the twitch responses of individual muscle fibers.

*2.3.1.7.1. Neuro-musculoskeletal glue*. The interactions between the neuromotor model and the musculoskeletal model are handled by a glue layer in NEUROiD. This layer calculates the activations (a value between 0.0 and 1.0) for the muscles in the musculoskeletal model using the spike train observed in the axons of motoneurons of corresponding cell groups. A model for proprioception is used to evaluate the afferent firing frequency using the muscle length and velocity at every time step.

Afferent feedback calculation is based on the quantitative approach suggested by Prochazka and Gorassini (1998). We use the equations described in Prochazka (1999) which are derived from Prochazka and Gorassini (1998) but normalized by the resting length of the muscle. Similar equations have been used in the calculation of afferent feedback in various models (Markin et al., 2010; Sreenivasa et al., 2013). The firing frequency of Ia afferents and II afferents are calculated using the below equations:

(1) $$\begin{array}{r} Ia_{Firing}=\alpha_{1}*v^{\gamma_{1}}+\beta_{1}*disp+\eta_{1}*act+base \end{array}$$

(2) $$\begin{array}{r} II_{Firing}=\beta_{2}*disp+\eta_{2}*act+base \end{array}$$

where *v* is the velocity of contraction and *disp* is the displacement of muscle, both normalized by the resting length of muscle. α_1_, γ_1_, β_1_, η_1_, β_2_, and η_2_ are constants whose values are derived from Prochazka (1999). *act* is the component of firing rate which is proportional to the alphamotoneuron activity, while the *base* represents the mean firing rate.

The equation highlights the power law relationship between the spindle afferent firing rate and rate of changes in the spindle length. We obtain the velocity and displacement from one of the musculoskeletal models described before and use them in the above equations to obtain the afferent feedback frequency. These afferent rates are used for stimulation of the interneurons and motoneurons, hence closing the control loop.

#### 2.3.2. Definition

The *curated model*, which is in a structured and concise form, can be used to generate a *model definition*.

Generation of simulator agnostic, structured model definition of the neuromotor system using the curated model is done by python scripts in NEUROiD (Figure 1B).

The model definition consists of a *core model* and some *auxiliary information*:

1. Definition of the core model is segregated into five sections: info, regions, segments, cell_groups, and net_connections. The info section contains meta information about the model (for example, name and viewing resolution). The segments section contains the details of spinal cord segments. The regions section contains all the 3D laminae in various spinal cord segments, while the cell_groups section holds the details about all the cell groups in the model. The net_connections section stores the details of network connections and their properties. Number of cells in a cell group, convergence ratio of neural connection and weight of synaptic connections are modeled as normal random variables in NEUROiD. Please refer to the Supplementary Material for further information on the structure of model definition.
2. Definition of *random cell positions* for cell placement in every cell group forms a part of auxiliary information. NEUROiD generates random positions (x, y, and z coordinates) inside the defined 3D region for every cell group. This ensures consistency in the 3D positions of cells displayed in visualization and the 3D positions used during simulation experiments.
  This setup allows Monte Carlo type of simulations to be performed with different randomly chosen cell positions in every simulation run. The distribution function used to generate cell positions is configurable. While uniform random distributions is supported currently, support for other distributions will be added in future.
3. Definition of *cell model templates* (in hoc file format) used for simulation forms another auxiliary information and it is generated by NEUROiD using the curated model. The curated model allows users to provide cell parameters (such as channel mechanism parameters, diameter, and length of section) as a random number. We use the NEURON Random() to generate normally distributed random numbers.
4. The contours of various laminae are translated with reference to a central canal region so that they are aligned along the neuraxis. NEUROiD uses these *translated contours* for simulation and 3D rendering.
5. Definition of *cell morphology* which is used to display the 3D structure of a cell also forms a part of model definition. NEUROiD stores the cell morphology information (derived from cell template definition) into a structured form suitable for visualization.

### 2.4. Simulation

The model definition is a simulator agnostic model representation. This is used to generate code that will be compiled and executed on simulation platforms. For the examples in the current paper, we run our simulations on NEURON (Hines and Carnevale, 1997). NEUROiD uses *Simulation Definition* from the user along with model definition to generate code for simulation. The simulation definition includes the parameters necessary for simulation such as duration of simulation, details of input stimulations to be provided and variables to be monitored during simulation.

The simulation definition can either be provided through the NEUROiD user interface (explained in section 2.5.1.1), or loaded from a saved file (explained in section 2.5.1.2), which stores the definition in a structured (json) format.

The model and simulation definition are used to generate the source code for the defined model in hoc format that can be simulated using NEURON. Python scripts are used to manage and monitor the simulation. These scripts create a hoc environment, load the model in hoc format, enable the input stimulations, record variables, store results into files, provide activation to the musculoskeletal model, calculate afferent feedback and close the sensorymotor loop. NEUROiD adopts a client-server architecture and performs compute and memory intensive simulation operations on a server machine with sufficient compute and memory resources (such as a high-performance computing cluster). The server runs on a dockerized ubuntu environment. Please refer to section 2.6 for more details on activities performed on the client and server side in chronological order during typical usage.

### 2.5. Visualization

**Figure 4:**
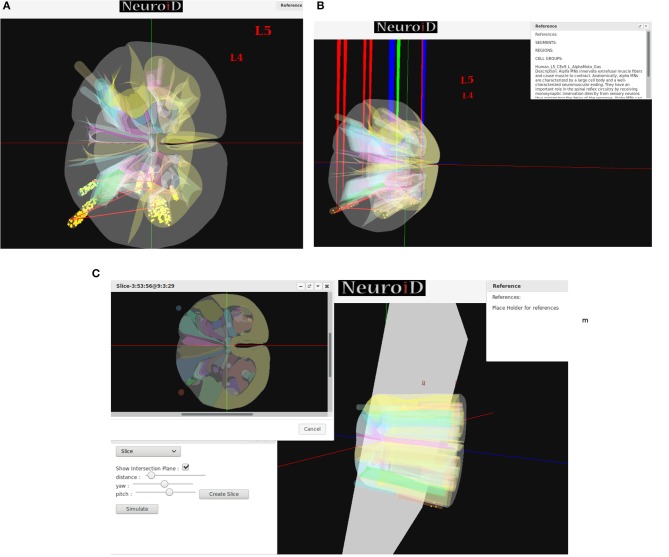
**(A)** The initial view of spinal cord rendered by NEUROiD. L4 and L5 sections of the cord are seen along the neuraxis in caudal-to-rostral direction. Various laminae boundaries (3D surfaces), cell groups (spheres) and the net connections (red lines) are seen. **(B)** NEUROiD allows users to inject current (red electrodes), measure current or voltage (blue electrode) and position an LFP electrode (green electrode). These can be placed manually, or a pre-defined setup in json format can be loaded using the “Save/Restore Setup” option to perform an experiment. LFP is evaluated using LFPSim (Parasuram et al., 2016) **(C)** NEUROiD allows users to slice the 3D model of spinal cord and visualize the laminae boundaries in 2D cross section at any point along the length of cord. The slicing plane can also be tilted to change the pitch and yaw angles before slicing.

NEUROiD uses a web-browser (client) for user interaction and visualization. An initial view of the spinal cord rendered on the web-browser is shown in Figure 4A. Note that the spinal sections, 3D boundaries of laminae, cells and net connections between cell groups are clearly visible. NEUROiD allows the user to zoom in, zoom out, translate, perform virtual electrophysiological operations such as placing current injection & measurement probes (section 2.5.1.1, Figure 4B), slicing (section 2.5.1.3, Figure 4C), viewing the results of experiments (section 2.5.1.4), and displaying stored literature references for the model (section 2.5.1.5, Figure 4B).

#### 2.5.1. Features

We describe the suite of features available in NEUROiD for user interaction, visualization, and virtual physiological experimentation.

##### 2.5.1.1. Current injection and measurement response

Injecting current at a specific cell or cell group is commonly used to study the electrophysiology of various cell types and circuits. NEUROiD provides a feature to inject current at any desirable location in the model. Since we render the 3D model of spinal cord on a 2D display, it is challenging to inject current at cell groups that are not rendered in the foreground. We solve this problem by listing all the cell groups that are intersected by the ray formed by a user click. User can select the specific region from the list where current injection is needed. Further the user can select either NetStim (a train of presynaptic stimuli in NEURON) or iclamp (current clamp in NEURON) and specify the amplitude of current, duration of current, initial delay in current injection and relevant parameters. The user can choose specific cell groups to view the response after a simulation. Similar to current injection, user can also select a cell group that is not present in the foreground but is intersected by the ray formed by a user click. The user can choose to record membrane current, membrane voltage or contributions of specific channel currents to be visualized in the response. NEUROiD displays a red electrode with the tip at the target injection cell group and a blue electrode for measurement (Figure 4B). LFP electrodes (in green) are also visible in the figure. NEUROiD allows users to specify large sets of injection and recording using json files. This is described below.

##### 2.5.1.2. Restore simulation definition

NEUROiD allows users to load a simulation setup saved in a structured (json) format. The json structure has five major sections: inputs, responses, setup, runs, and plots. The inputs section defines the various simulation inputs that the user intends to provide for simulation. This includes the type of input (for example, iclamp, NetStim) and cell groups to which the input is provided. The user can provide arbitrary functions to describe the input magnitude using piecewise linear approximations. The responses section defines the model parameters that the user wants to record during simulation. This includes the type of parameter to record (for example, voltage, current, spike) and cell groups to record from. The plots section is used to define the parameters to be plotted and type of plots the user would like to view. This design where the measurement of responses and their plots are separated, allows us to record as many simulation variables as needed and save them in files, and use them later for plotting and post-processing. The runs section is used to define the parameters that control the simulation (for example, duration of simulation, the piecewise linear inputs). Please refer to the Supplementary Material for further information on the structure of simulation definition json.

##### 2.5.1.3. Slice

Since their first description by Rexed (1954), the concept of laminae has been very popular for describing cytoarchitectonic boundaries in the spinal cord. The laminae are organized in a series of layers from dorsal to ventral, lamina 1 being the most dorsal. The structure, cytoarchitecture, and chemoarchitecture for each lamina is summarized in Sengul et al. (2012). The slicing feature in NEUROiD allows the users to slice the model at a specific location and view the cross section in an inset (Figure 4C). The slicing can be done anywhere along the length of the model (neuraxis). Further, the slicing plane can be tilted along the x (yaw) and/or y (pitch) axis to view the slice at a specific angle.

##### 2.5.1.4. Plotting

NEUROiD plots the results of experiment in javascript dialog windows. Primary variables (for example, membrane potential, spike train) or secondary evaluated variables (for example, frequency-current curve, spike rate adaptation curve) can be plotted. The user can specify the list of variables to be plotted in the simulation definition file. NEUROiD uses plotly ^3^ library for plotting, which allows users to view, edit, and analyze the plots online.

##### 2.5.1.5. Reference

NEUROiD is an easily referenceable collection of relevant information curated from literature. Quite often, models are viewed with skepticism as they are not wholly transparent with respect to the parameters that went into their creation. To ensure transparency and traceability, NEUROiD includes a feature to store and display all the references used for creating the model. When the user performs a right click on a specific point on the spinal cord model, the references window (top right in Figure 4B) shows the references used for the specific model element. This information is parsed from the curated model which collates all the references that went into the curation process.

Further details on the features and organization of NEUROiD can be found in Supplementary Material and the documents available along with the source code.

### 2.6. Typical Usage

**Figure 5:**
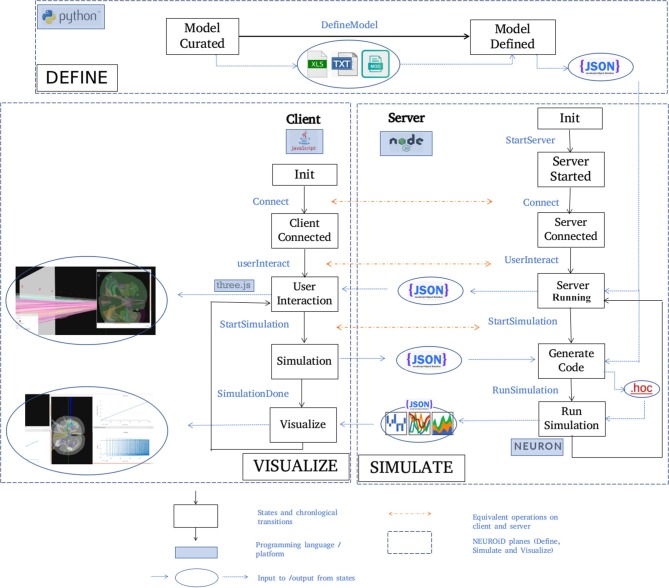
Typical workflow in NEUROiD. In the *Define* plane, the “model curated” state indicates the completion of manual curation of data into xls, txt and mod files. This is then converted to json model definition using python scripts and the state changes to “Model defined.” A NodeJS server is started in the *Simulate* plane and waits in the “server started” state for connection request from a client. On the *Visualize* plane, a client (written in javascript) tries to connect to the server upon user initiation. Upon successful connection, the client and the server enter the “Server connected” and “Client connected” state respectively. The server sends the model definition to the client which renders the 3D model and performs user interaction. Upon a simulation request from the user, the client enters the “Simulation” state and the server enters the “Generate code” state to generate the hoc code. This hoc code is used to perform simulation with NEURON backend. The results of the simulation are sent back to the client for visualization.

A typical set of activities performed on NEUROiD are shown here (Figure 5) in chronological order. The *model definition* is generated from the *curated model*. NEUROiD uses a *NodeJS server* ^4^ to perform all server-side operations. The node server waits for connection request from a client (a web-browser such as firefox^5^). On receiving the connection request, the node server sends the *model definition* to the connected client. The client uses the *model definition* to render a 3-dimensional view of the neuromotor system, using the threejs ^6^ javascript library. An initial view of the spinal cord rendered by the client is shown in Figure 4A. The client also allows the user to *interact with the model*. In section 2.5.1, we have described some of the client-side features offered by NEUROiD in more detail. The client also allows the user to specify a *simulation definition* (current injections to be made, voltage/currents to be measured, plots to be displayed). Details of such setup are provided in Supplementary Material. The server then *generates code* (in hoc^7^ format) using the *model definition*, and the *simulation definition* sent by the client. Generated code is used by NEURON (Hines and Carnevale, 1997) simulation environment to simulate the experiment(s) requested by the user on the defined model. The results of simulation are then sent back to the client for rendering on the web-browser.

### 2.7. Model Integration in NEUROiD

One of the core philosophies of NEUROiD is to create a user-friendly platform for model definition by curation and combination of multiple existing models. The *integrated model* created from multiple *constituent models* should exhibit the properties of interest from all the constituent models. Creation of such a model is a non-trivial task requiring comparison of multiple models, exploration, and tuning of model parameters.

**Figure 6:**
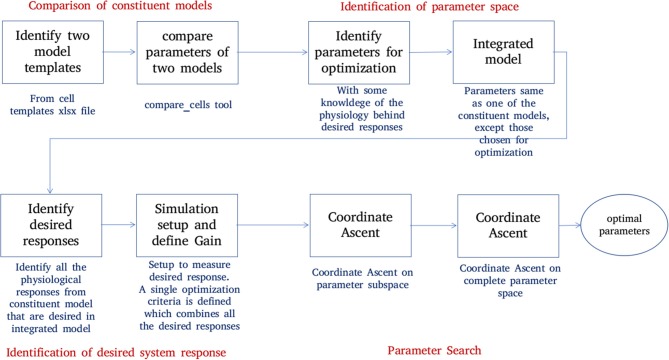
Creation of an integrated model. Constituent models used for creation of an integrated model are identified and compared. A set of parameters responsible for the key features of constituent models are identified. The desired system response and objective function are identified. The parameter space is divided into subspaces. Coordinate ascent is performed on each parameter subspace with an aim to achieve the desired system response on the integrated model.

Figure 6 shows the process of parameter search and creation of an integrated model at a high level. We use a *coordinate ascent* based approach to explore for optimal parameters in the integrated model. Coordinate descent (Wright, 2015) is a widely used parameter search algorithm in machine learning, control systems (Luo and Tseng, 1992; Patrascu and Necoara, 2015; Chen et al., 2017) and recently in gene selection methods (Ghalwash et al., 2016). Though such parameter search algorithms provide optimum parameters for a convex optimization function, they are known to converge to a local optimum (and not global optimum) for high dimensional non-linear error surfaces. This is generally acceptable because recovering the global minimum becomes harder as the dimensionality of the error hypersurface increases and the global minimum may lead to overfitting (Choromanska et al., 2014; Pascanu et al., 2014).

The process of creating an integrated model involves the following steps:

1. **Comparison of constituent models:**
  NEUROiD stores all model information in a structured and tabulated form. This allows for an efficient comparison between two models. NEUROiD provides a tool (called compare_cells) that shows the difference between two constituent cell models in a tabulated, clear and concise format. This allows the users to identify the parameters from constituent cell models that should be imported and tuned in the integrated model. Currently, this is a command line tool which compares two cell types. In future releases, this tool will be integrated to show the output on the web-browser and compare models at Meso and Macro scales as well.
2. **Identification of parameter space:**
  An integrated cell model is created based on the cell parameters of one or more of the constituent models. Based on the output of compare_cells and the *properties of interest* in each of the constituent models, a set of parameters is manually chosen. These parameters are the ones which most likely define the properties of interest in the constituent models. The goal now is to explore and identify values for these parameters such that the integrated model displays the properties of interest observed in all the constituent models along with some emergent properties.
3. **Identification of desired system response**:
  In order to perform automatic parameter search on any system, it is essential to define the desired system response and a corresponding objective function. The desired system response is the ideal output of the system for a given input. The objective function measures how close the system response (with current parameter set) is, to the ideal system response. Hence, the goal of parameter search algorithm is to now converge to a set of parameters for the integrated model which maximizes the objective. For the integrated model, the desired system response could include one or more of the micro, meso, or macro level properties identified in constituent models. The objective function is defined as the sum of Pearson's correlation coefficients between current system response and each of the constituent model responses. The parameters for the integrated model are typically identical to one of the constituent models, except for the parameters that are identified in step “identification of parameter space” above.
4. **Parameter search:**
  With a desired system response and an objective function, the parameter space is explored to find the set of parameters that maximizes the objective function.
  Finding an analytical expression to indicate how the objective function changes with respect to each of the search parameters is difficult for a complex and non-linear system such as the one we are dealing with now. It becomes even more challenging in our case where we are interested in finding a set of parameters that should provide multiple results from the same model.

Our Coordinate ascent search has two aspects:

- The direction and magnitude of change in the parameter hyperspace depends on the amount of correlation with ideal results. For every set of parameters that the algorithm proposes, the constituent system responses and the objective function are evaluated. The parameters are changed, few at a time to gradually approach an optimum set of parameters.
- We perform this coordinate ascent on the individual parameter subspaces and obtain a few seed points. These are then used to perform search in the hyperspace formed by the set of all the parameters.

## 3. Results

In section 3.1.1, we describe a simple motoneuron model built in NEUROiD based on parameters from a published model (Courtine et al., 2016). We then build a system level neuromotor model of L4 and L5 segments of spinal cord. Instances of alphamotoneuron cells in this system model use the motoneuron model that was previously created. In section 3.1.2, we describe how our model is validated. In section 3.1.2.1, we validate its single cell neurophysiological properties (frequency-current curve). The system level property (spinal reflex recruitment curve) is validated in section 3.1.2.2 by comparing with the results from published model. In section 3.1.2.3, we describe integration of the neuromotor model with an OpenSim musculoskeletal model. In section 3.2.1, we describe additional cell models built in NEUROiD based on parameters from published literature (Booth et al., 1997; Destexhe, 1997; Cisi and Kohn, 2008). The single cell neurophysiological properties of these cells are validated in sections 3.2.2.1–3.2.2.3 by comparing the properties of reference models. In section 3.2.2.4, we validate the orderly recruitment of motoneuron cell types in the neuromotor model by comparing the rheobase of cell models with published values. In section 3.2.3, we describe the construction of an integrated cell model in NEUROiD and validation of its single cell neurophysiological and system properties by comparing with the properties of constituent cell models.

### 3.1. Simple Motoneuron Model

We built a motoneuron cell model based on parameters from Courtine et al. (2016). The cell model was validated by comparing single cell neurophysiological properties of the model with the reference model. This motoneuron cell model was used in a spinal cord model of L4 and L5 segments in NEUROiD. Meso scale properties of the neuromotor model were validated by comparing the spinal reflex recruitment curves of the model with the reference. We also demonstrate the integration of OpenSim musculoskeletal model with NEUROiD.

#### 3.1.1. Model Definition and Generation

We defined the cell sections, topology, and ligand gated channel properties of the motoneuron cell model derived from Courtine et al. (2016) and populated the appropriate input xlsx file in NEUROiD. This forms the Micro/Anatomy property of the model. We defined the soma, inseg (axon hillock), node, paranode, and dendrite sections along with the corresponding channel mechanisms and parameters. We set the soma diameter to be a random variable with a mean of 82 μm, to match the published values for slow type motoneuron (Fleshman et al., 1981). The topological connections between the sections were also derived from reference and defined along with the ligand gated channels and their parameters. The mod files for the channel mechanisms form the Micro/Physiology component of the model. These are obtained from ModeldDB (Hines et al., 2004).

To validate the network properties of our model with the reference, we developed two different models for the ankle joint along with its controlling neural circuitry. These models are described below:

1. **Native Ankle Model:**
  We now show an example of musculoskeletal model definition using native NEUROiD interface. A biomechanical model for the specific musculoskeletal subsystem is identified and the governing differential equations are obtained. These equations can then be coded as mod files which can be solved by NEURON along with rest of the neuromotor system.
  The default ankle model consists of a rigid body model of the foot (Figure 3A). *R*, *O*, and *Q* represents heel, hinge, and toe, respectively. *PQ* and *PR* represent lengths of tibialis anterior and gastrocnemius muscles respectively. The ankle makes an angle θ with the positive *X* axis. As the forces along the muscles change, the ankle rotates about the origin *O*. The governing differential equation that determines the configuration of ankle is as follows:
  (3) $$\begin{array}{r} M=I\ddot{\theta } \end{array}$$
  Where *M* is the moment of forces (due to contraction of tibialis anterior and gastrocnemius muscles) about *O*, *I* is the moment of inertia and θ is the angle made by the ankle with the positive *X* axis (Figure 3A).
  This model is implemented as a mod file so that the ankle angle is solved using NEURON solver at every step of simulation.
  The Meso/Biomechanics component consists of the transfer function between the motoneuron activation and the change in muscle properties.
  In the default model, an action potential from the motoneuron is approximated using δ(*t*), which is the unit impulse function. Force produced by motor units is modeled as the output of a linear time invariant system whose unit impulse response is the twitch response (Fuglevand et al., 1993; Cisi and Kohn, 2008).
  The twitch response of a motor unit to an action potential in associated motoneuron forms the Micro/Biomechanics component of the model.
  At every timestep, the ankle angle (θ), length of tibialis anterior (*PQ*), and gastrocnemius (*PR*) muscles are obtained.
2. **OpenSim Model:**
  The lower body mechanics model (Delp et al., 1990) was integrated with NEUROiD. This model (Figure 3B) internally implemented all the aspects (Micro, Meso, and Macro) of the biomechanical model.

We define a model of the 4th and 5th lumbar segments of the spinal cord that has the cell groups and synaptic connections (derived using muscle synergies) responsible for ankle movement.

Tibialis anterior and gastrocnemius are the dominant muscles for ankle dorsi-flexion and plantar-flexion respectively. The muscle synergy information for these muscles are updated. The anatomical localization (Sharrard, 1955) and properties of associated motoneurons in lamina-9, excitatory, and inhibitory interneurons in lamina-6 and afferent neurons in dorsal root ganglion are updated in neuronal cell types table. The synaptic connection rules between the defined cell groups are updated.

**Figure 7:**
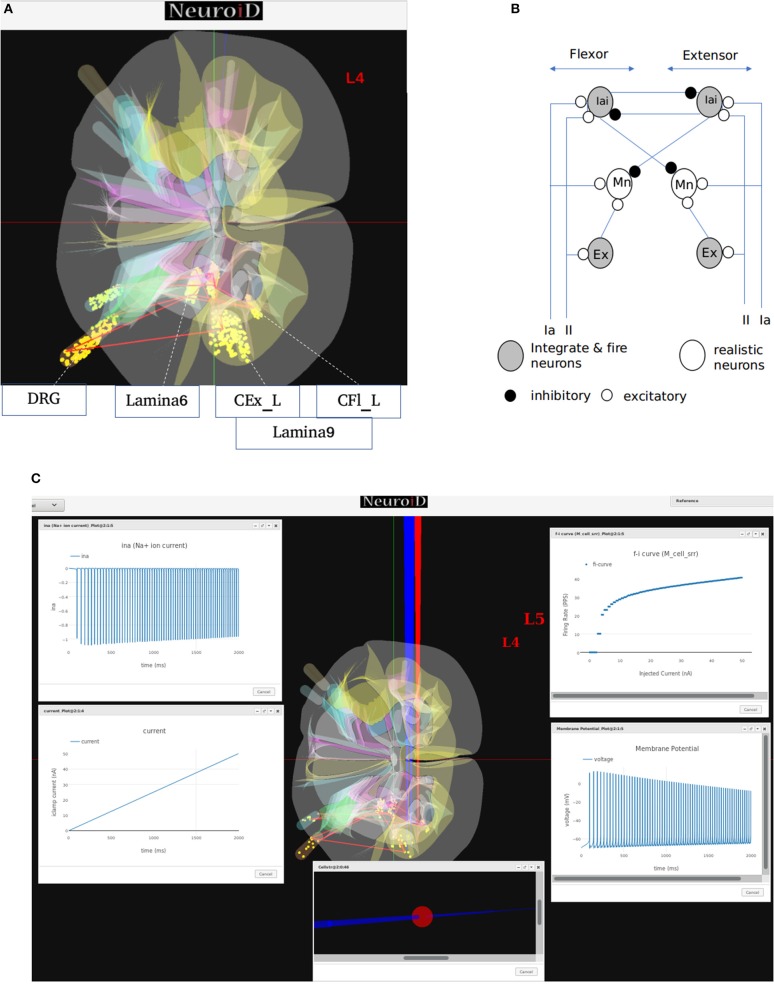
**(A)** This figure shows the network and system level model description of the model used for all systems level experiments. The various motoneuron, interneuron, and dorsal root ganglion cell groups placed within their respective 3D laminae boundaries can be seen here. The net connections between different cell groups are also shown as red line. **(B)** The representation of the feedback circuit and net connections for the flexor and extensor group used in simulation to obtain spinal reflex recruitment curves, orderly recruitment and integration with OpenSim. **(C)** A Typical view of output screen in NEUROiD after simulation. The 3D model of spinal cord with the current injection probes (red) and the measurement probes (blue) is seen in the background. The experimental results are plotted in small windows on the screen. Here, we see the FI-curve of M_cell_srr (top-right). We see the firing frequency increase with an increase in the amplitude of injected current for a ramp input current (bottom-left). The membrane potential at the soma (bottom-right) is also recorded and plotted. The cell morphology for the motoneuron is defined by the user in the cell template definition xls file. This information is used to render a 3D model of the cell with detailed morphology (bottom-center). The soma is shown as a sphere in red while the axon and dendrite sections are shown as cylinders in blue. We can also record the channel mechanism currents in NEUROiD. The *Na*^+^ ion current is recorded and plotted (top-left) in this example. The simulation definition file is used to specify all the parameters that need to be recorded and plotted.

The generated motoneuron cell groups (Figure 7A) are placed in the left crural extensor (CEx_L) laminae in lumbar section 5 and in the left crural flexor laminae (CFl_L) in lumbar section 4. The generated afferent neuron cell groups are the IaAfferent and IIAfferent neuron groups in the dorsal root ganglion region. IaInterneuron and excitatory interneuron cell groups in the laminae-6 of spinal gray matter are also generated in both L4 and L5 sections.

In our simulations, we modeled 169 alpha motoneuron cells, 60 Ia and II afferent cells each, and 400 interneurons in each segment of the spinal cord (Courtine et al., 2016). The membrane potential at soma of each alpha motoneuron cell is described using a modified Hodgkin-Huxley model. It consists of sodium, calcium activated potassium, N-type and L-type calcium channels (McIntyre et al., 2002). The interneurons were modeled as integrate-and-fire cells.

From the derived synaptic connections (Figure 7B), we can see that the afferent fibers form excitatory connections with agonist motoneurons, excitatory, and inhibitory interneurons. Ia inhibitory interneurons form inhibitory connections to antagonist motoneurons while the excitatory interneurons form excitatory connections with agonist motoneurons (Pierrot-Deseilligny and Burke, 2012). These synaptic connections are also shown as red lines between cell groups in Figure 7A.

We call this cell model M_cell_srr, since we plan to reproduce *spinal reflex recruitment* curves from this model.

#### 3.1.2. Simulation and Visualization

Here we describe selected single-cell neurophysiological experiments performed on the created cell model.

Frequency-current (FI) relationships of motoneurons demonstrate the active properties of the cell for suprathreshold current injections. Step current injections have been used to study the instantaneous firing frequency adaptation (Granit et al., 1963; Kernell and Monster, 1981) while triangular current injections have been used to study effect of persistent inward currents and plateau potentials (Booth et al., 1997; Kiehn and Eken, 1998; Lee and Heckman, 1998) in motoneurons.

NEUROiD allows users to start a simulation and visualize the results on the client (web-browser). Figure 7C shows a typical output screen on NEUROiD. After completion of an experiment, results are plotted in small windows. The results of a simulation performed to obtain the FI curve of M_cell_srr are shown here. The FI curve (top-right), membrane potential (bottom-right), current due to specific channel mechanism (top-left), cell morphology (bottom-left), and injected current (bottom-center) can be seen in the figure.

##### 3.1.2.1. FI (Frequency-Current) curve

This experiment involved stimulating the cell model with an increasing ramp current and measuring the instantaneous firing frequency. The current input is defined as a linear piecewise part in the "inputs" section of the experiment setup json file. The current ramps up from 0 nA amplitude at 0 ms to 50 nA at 1,000 ms. It is known that the rheobase of the FF type motoneuron is highest (17.5–25.1 nA) and that of the S type motoneuron is the lowest (3.5–6.5 nA), while the FR type has a rheobase that lies in between the two (6.5–17.5 nA) (Fleshman et al., 1981; Cisi and Kohn, 2008). Fifty nanoamperes is well above the threshold current needed for all the three types of motoneurons to fire.

**Figure 8:**
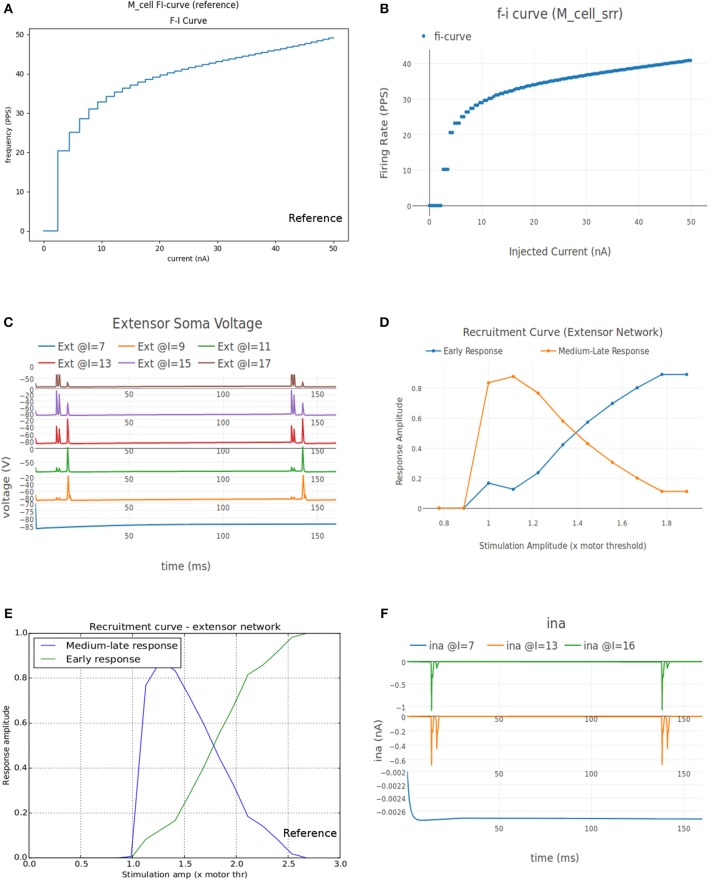
**(A)** FI curve of the reference motoneuron cell model derived from Courtine et al. (2016). **(B)** FI curve of *M*_*cell*_*srr*, a motoneuron model in NEUROiD. The rheobase of this model is close to 3nA, same as the reference model in **(A)**. **(C)** The extensor soma voltage for various EES stimulations, averaged over all the cell instances in the cell group. It is seen that the early response activation (first set of spikes after 125 ms) increases with increase in EES stimulation magnitude, but the medium-late response (second set of spikes after 125 ms) first increases and then decreases. The bottom most plot (shown in blue) in the figure is obtained for an EES stimulation of 7 mA. The stimulation amplitude is increased in steps of 2 mA to obtain other plots. **(D)** Spinal reflex recruitment curves elicited by EES Stimulation of the extensor network simulated in NEUROiD. The medium-late response reduces to less than 20% of its peak value when input stimulation is twice the motor threshold. **(E)** Spinal reflex recruitment of extensor group, reproduced from Courtine et al. (2016). The early and medium-late response is plotted here. Similar to **(D)**, we see that the medium-late response reduces to less than 20% of its peak value when input stimulation is twice the motor threshold. **(F)** The *Na*^+^ ion current is recorded and plotted for various EES stimulations.

Figure 8A shows the FI curve that was obtained with the reference motoneuron model from Courtine et al. (2016). We see that the FI curve displays type 2 dynamics and the rheobase matches with the typical value expected for a S type motoneuron (Cisi and Kohn, 2008, Table 2). The FI curve for M_cell_srr is shown in Figure 8B. We note that this is very similar to the reference FI curve in (a).

##### 3.1.2.2. Spinal reflex recruitment curve

This experiment aims to validate the meso scale properties of the motoneuron model and spinal network created in NEUROiD. For this, we define “EES” type input to stimulate the cell groups in the extensor network. The setup was defined to run 12 different experimental runs each for a duration of 160 ms and for different EES current amplitude. The first experimental run used a current of 7μA as input and the subsequent experiments increased the input current by 1μA each. The EES frequency was set to 8Hz with a delay of 10 ms.

The average soma membrane potential in extensor motoneuron group is shown in Figure 8C. Since the EES is set to have a frequency of 8 Hz with a delay of 10 ms, the first input pulse is triggered at 135 ms. The responses observed with a latency of 9–11 ms, 4.5–5 ms, and less than 3 ms are considered as *late, medium* and *early* respectively (Gerasimenko et al., 2006). The medium-late responses are observed due to activation of monosynaptic and disynaptic pathways in reflex circuit, while the early response is due to direct recruitment of motoneurons (Capogrosso et al., 2013). We observe that neither the medium-late nor the early response are not seen below a threshold (10 mA). Above the threshold, medium-late responses increase rapidly and then decrease as the input stimulation intensity is increased, while the early response increases with increase in stimulation intensity monotonically until all the fibers are recruited. The spinal reflex recruitment curves elicited by EES stimulation for extensor network are show in Figure 8D. The stimulation amplitude is defined in terms of motor threshold, which is the minimum magnitude of input stimulation for which the early response is observed. We also observe that the medium-late response came below 20% of its maximum value when the stimulation amplitude was twice the motor threshold. The spinal reflex recruitment curves of extensor network observed in Courtine et al. (2016) are also shown in Figure 8E for comparison. We observe again that the medium-late response falls below 20% of its maximum for a stimulation amplitude that is twice the motor threshold.

To gain more insight into the spinal reflex recruitment curves, we plotted the individual component currents due to channel mechanisms adopted (McIntyre et al., 2002). We see that the inward sodium current (ina) (Figure 8F) and outward delayed rectifier *K*^+^ (ikrect) show the behavior that leads to the recruitment curves. Though linear leakage (il), N-type *Ca*^2+^ (icaN), and L-type *Ca*^2+^ (icaL) show the same pattern, they are an order of magnitude smaller and hence, unlikely to have any major effect. This agrees with the observation in Capogrosso et al. (2013) that sodium current is a major contributor in medium and late responses. These additional plots were obtained by specifying the new parameters to be recorded and plotted in the setup json file. No other change in the simulation setup or model were required.

##### 3.1.2.3. Integration with OpenSim

In this experiment we demonstrate the integration of NEUROiD with an external musculoskeletal simulator such as OpenSim (Seth et al., 2011). A lower body mechanics model (Delp et al., 1990), which modeled the tibialis anterior and gastrocnemius muscles was used. The firing rate of the action potential spike train of the flexor and extensor motoneuron group from NEUROiD was converted to activation values as expected (a value between 0.0 and 1.0) by the OpenSim model. For every simulation step, the activation values were sent to the OpenSim model. After a single step of Opensim simulation, the updates in muscle length and velocity were used by NEUROiD to calculate the afferent feedback as suggested by Prochazka and Gorassini (1998), hence controlling the ankle muscles in a biologically realistic closed loop.

We define two inputs of type “EES” (implemented using NetStim in NEURON), one each for the flexor and extensor motoneuron group. The extensor network is stimulated between 0 and 60 ms, while the flexor network is stimulated between 130 and 160 ms from start of the experiment.

**Figure 9:**
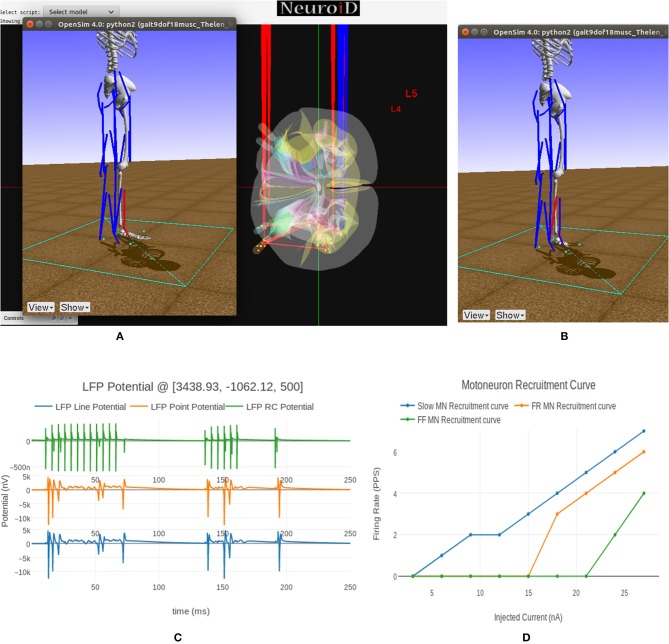
**(A)** This figure shows NEUROiD and OpenSim integration. The OpenSim model is driven by the motoneuron activations evaluated in NEUROiD and the calculated afferent firing rates are used by NEUROiD as a proxy for afferent stimulation (refer Figure 3). The OpenSim model is seen performing a dorsi-flexion when the flexor motoneurons (tibialis anterior) were activated during simulation. **(B)** OpenSim model performing plantar-flexion when the extensor motoneurons (gastrocnemius) were activated during simulation. **(C)** An LFP probe was placed at a point close to the L4 segment of spinal cord during the simulation. The 3D location of the probe was (x:3438, y:–1062, z:500). This figure shows the plot of recorded LFP. LFP is evaluated using the LFPSim (Parasuram et al., 2016), which is integrated into NEUROiD. LFPSim evaluates LFP using point source approximation, line source approximation and low pass RC methods. LFPSim uses the *extracellular* mechanism available in NEURON to simulate extracellular potentials. We used the default values of extracellular capacitance (xc), extracellular resistance (xaxial) and extracellular conductivity (xg) set by LFPSim. **(D)** This figure shows the orderly recruitment of motoneuron groups. We see that the S type motoneurons start firing at 3 nA. The FR and FF type motoneurons are recruited at 15 and 25 nA, respectively. These thresholds match with the rheobase values of slow, fast fatigue resistant and fast fatigue motoneuron models (refer Cisi and Kohn, 2008, Table 2).

We see the flexor muscle being activated during the simulation in Figure 9A thereby causing a dorsi-flexion, while Figure 9B shows the extensor being activated and causing a plantar-flexion.

In Figure 9C we show the LFP evaluated during this experiment when the electrode was placed close to the motoneuron group in the L4 segment of spinal cord. LFPSim evaluates LFP using point source approximation, line source approximation and low pass RC methods. LFPSim uses the *extracellular* mechanism available in NEURON to simulate extracellular potentials.

**Figure 10:**
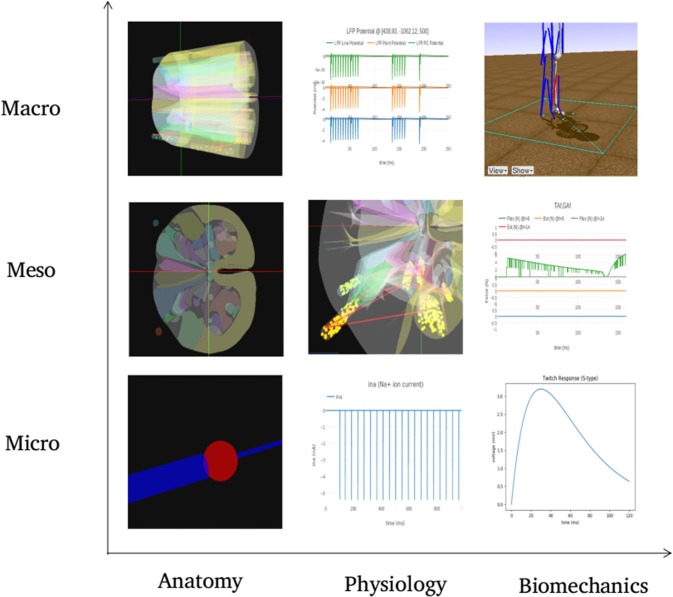
The figure shows snapshots from various simulations in NEUROiD representing the 9 cubelets of the *visualization* plane. In the *Macro*/*Anatomy* cubelet, we see the 3D model of L4 and L5 segments of spinal cord. Contours in the sliced L4 segment are seen in the *Meso*/*Anatomy* cubelet. The cell morphology that forms the *Micro*/*Anatomy* component, can be seen in the corresponding cubelet. In the *Macro*/*Physiology* cubelet we see the local field potential evaluated at a user specified point in 3D space. The *Meso*/*Physiology* cubelet shows the synaptic connections and neural circuits that we have defined in the L4 and L5 segments. Ionic currents due to channel mechanisms forming the *Micro*/*Physiology* component is seen in the corresponding cubelet. Along the biomechanics discipline, the ankle model and angle made at the ankle joint are seen in the *Macro*/*Biomechanics* cubelet. Force generated in muscles as a response to the motoneuron activation is seen in the *Meso*/*Biomechanics* cubelet. The *Micro*/*Biomechanics* component is defined by twitch response of a muscle fiber is also seen.

Figure 10 summarizes visualization of the 3x3 cubelets across scales and disciplines for the experiments performed on the example neuromotor model. In the Macro/Anatomy cubelet, we see the 3D model of L4 and L5 segments of spinal cord. Contours in the sliced L4 segment are seen in the Meso/Anatomy cubelet. The cell morphology that forms the Micro/Anatomy component, can also be seen. In the Macro/Physiology cubelet we see the local field potential evaluated at a user specified point in 3D space. The Meso/Physiology cubelet shows the synaptic connections and neural circuits that we have defined in the L4 and L5 segments. Ionic currents due to channel mechanisms forming the Micro/Physiology component is seen in the corresponding cubelet. Along the biomechanics discipline, the ankle model and angle made at the ankle joint are seen in the Macro/Biomechanics cubelet. Force generated in muscles as a response to the motoneuron activation is seen in the Meso/Biomechanics cubelet. The Micro/Biomechanics component is defined by twitch response of a muscle fiber.

Such a visualization may also influence the choice of cell models to achieve specific network or system level behaviors.

### 3.2. Integrated Cell Model

Our goal is to build an integrated cell model that can reproduce properties of constituent cell models. For this, we first build additional cell models in NEUROiD. We validate these models by comparing the neurophysiological properties with reference. We then build an integrated cell model using techniques described in section 2.7.

#### 3.2.1. Additional Cell Models - Definition and Generation

We created additional motoneuron cell models in NEUROiD and validated their neurophysiological properties. Firstly, we defined a model based on Destexhe (1997) and called it M_cell_Slow. Being an integrate-and-fire model, this had lesser computational complexity compared to the previous model. The Micro/Anatomy and Micro/Physiology properties were populated in relevant xlsx files as done for the previous model. We also added fast fatigue resistant (M_cell_FR) and fast fatigable (M_cell_FF) motoneurons in a similar manner. The electrotonic parameters for these cell types were specified based on Cisi and Kohn (2008).

It is known that in presence of certain ion channel blockers or neurotransmitters, more complex firing patterns can be evoked in motoneurons. For a ramp current input, the action potential firing frequency increases with increasing current injection. Due to increase in average dendritic voltage, a dendritic plateau potential is achieved that causes the action potential firing frequency during the descending input ramp to be higher than the corresponding value during the ascending phase. Such a bistable firing pattern endows motoneurons with a mechanism for translating short lasting synaptic inputs into long-lasting motor output (Hounsgaard and Kiehn, 1989). This firing behavior is explained by activation of a plateau potential mediated by an L-like *Ca*^2+^ current. A computational model of motoneurons displaying the bistable firing pattern is discussed in Booth et al. (1997). We implemented a motoneuron cell model in NEUROiD based on parameters from Booth et al. (1997) and called it M_cell_bistable.

#### 3.2.2. Additional Cell Models - Simulation and Visualization

##### 3.2.2.1. FI (Frequency-Current) curve

The experiment setup for FI curve is same as the setup used to obtain FI curve of M_cell_srr.

**Figure 11:**
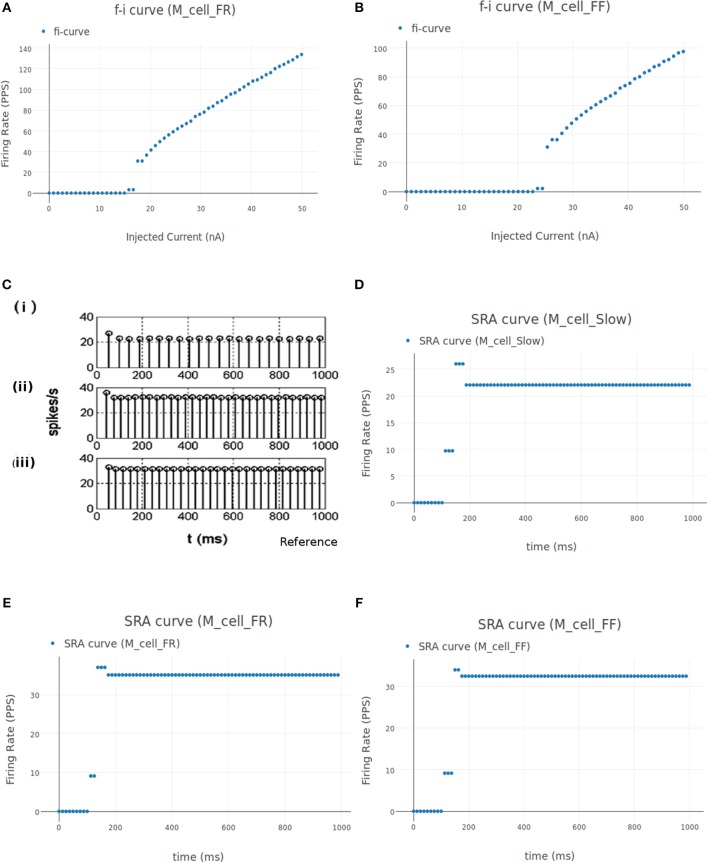
**(A,B)** FI curve of *M*_*cell*_*FR* and *M*_*cell*_*FF* motoneuron cell models in NEUROiD. The rheobase from these plots matches with the values listed in Cisi and Kohn (2008) (Table 2). **(C)** The spike rate adaptation of Slow, FR and FF type motoneurons [(i), (ii), and (iii), respectively], reproduced from Cisi and Kohn (2008). **(D)** Spike rate adaptation curves of Slow type motoneuron model in NEUROiD. The basal firing rate of 22 Hz is achieved within a couple of inter spike intervals. This matches with the subplot (i) in **(C)**. **(E,F)** Spike rate adaptation curve for FR and FF type motoneuron, respectively. Basal firing rate of 32 Hz is achieved with a stimulation of 18 nA for FR type motoneurons and 25 nA for FF type motoneurons. Similar to subplot (ii) and (iii) from **(C)**, the basal firing rate is achieved within a couple of inter spike intervals.

Figures 11A,B shows the FI curve of M_cell_FR and M_cell_FF respectively. We see that the FI curves displays type 2 dynamics and the rheobase matches with the typical value expected for FR (fast fatigue resistant) and FF (fast fatigue) type motoneurons (Cisi and Kohn, 2008, Table 2).

##### 3.2.2.2. SRA (Spike Rate Adaptation) curve

This experiment involved stimulating the cell model with a step current and measuring the instantaneous firing frequency. Step input is defined as a piecewise linear approximation with value 0 nA upto 100 ms and an amplitude greater than the firing threshold of the cell, thereon upto 1,000 ms. The same is defined in experiment setup json file in the “inputs” section.

The spike rate adaptation (also called firing rate adaptation) curves of Slow, FR and FF type motoneuron models was obtained in Cisi and Kohn (2008) and reproduced in Figure 11C. Figure 11D shows the firing rate adaptation curve of Slow type motoneurons obtained in NEUROiD. The basal firing rate of 22 Hz for the Slow type motoneurons is achieved within a few interspike intervals. This can also be observed in the reference plot (subplot (i) of Figure 11C) An input stimulation current of 12 nA was used for this simulation. Similar behavior is observed with FR (Figure 11E) and FF (Figure 11F) type motoneurons. A basal firing rate of 32 Hz is achieved with a stimulation of 18 nA for the FR type motoneurons and 25 nA for the FF type motoneurons. Thus, the results obtained on NEUROiD are similar to the spike rate adaptation plots in Cisi and Kohn (2008).

##### 3.2.2.3. Bistable firing

**Figure 12:**
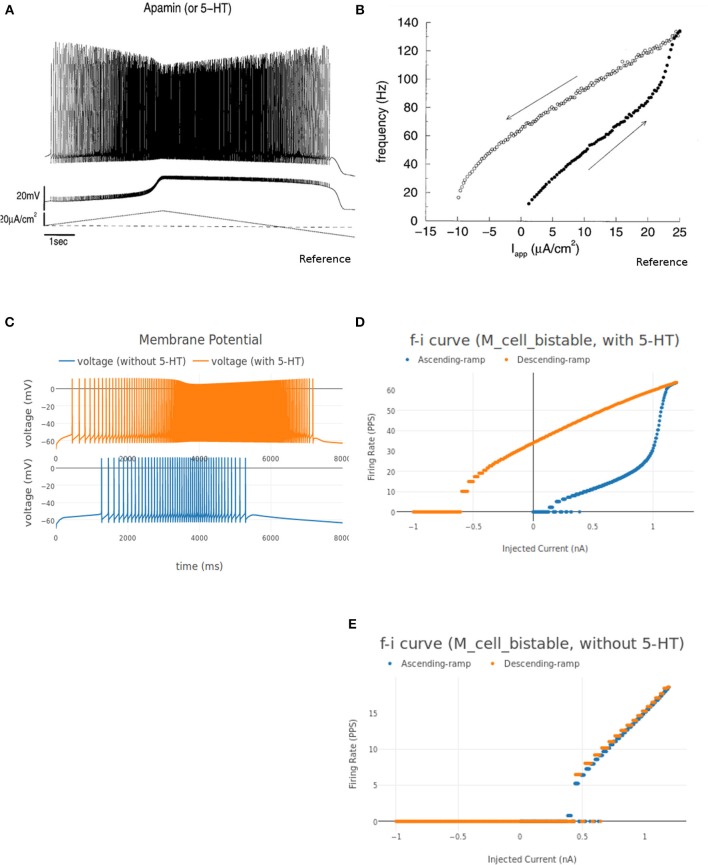
**(A,B)** Membrane potential at soma of the motoneuron and instantaneous FI curve of motoneuron (with 5-HT) respectively, reproduced from Booth et al. (1997). **(C)** The membrane potential recorded at soma for an increasing followed by decreasing ramp input current for *M*_*cell*_*bistable* in NEUROiD (with and without 5HT). **(D–E)** The instantaneous FI curve for an increasing followed by decreasing ramp input current of *M*_*cell*_*bistable* in NEUROiD (with and without 5 HT respectively).

The instantaneous firing frequency plot for ascending and descending ramp current and membrane potential plot from Booth et al. (1997) is reproduced in Figures 12A,D, respectively. Figure 12B shows the instantaneous firing frequency with 5-HT (serotonin receptors) enabled and Figure 12C shows the instantaneous firing frequency with 5-HT disabled for M_cell_bistable. we enable the 5-HT by reducing the gkcabar_KCa parameter by 40% (Cisi and Kohn, 2008). The membrane potential at the soma of M_cell_bistable is shown in Figure 12E. The results obtained in NEUROiD are similar to the bistable firing plots in Booth et al. (1997).

##### 3.2.2.4. Orderly Recruitment

It is known that motoneuron recruitment is size-ordered (Henneman et al., 1965). The smaller, low force, long endurance slow (S-type) motoneurons are recruited first, while the larger FR and FF type motoneurons are recruited when larger muscular forces are needed for shorter durations of time (Purves et al., 1989). This behavior is also observed in the motoneurons contributing to the human H-reflex (Buchthal and Schmalbruch, 1970). Table 2 in Cisi and Kohn (2008) summarizes the typical values for size, rheobase and other parameters of S, FR and FF type motoneurons, while Table 3 summarizes the twitch and force properties for the three types of motoneurons.

We define an input of type “iclamp” to stimulate the S, FR, and FF motoneuron cell groups directly, representing the cumulative stimulus to the motoneuron cell groups. The iclamp input is varied from 3 to 30 nA in steps of 3 nA. The recruitment plot is shown in Figure 9D. We see that the S, FR, and FF type motoneurons start firing at 3, 15, and 25 nA, respectively, matching their respective rheobase values (Fuglevand et al., 1993).

#### 3.2.3. Creation and Validation of Integrated Cell Model

We created an integrated motoneuron cell model using the techniques mentioned in section 2.7 and called it M_cell_Integrated.

**Figure 13:**
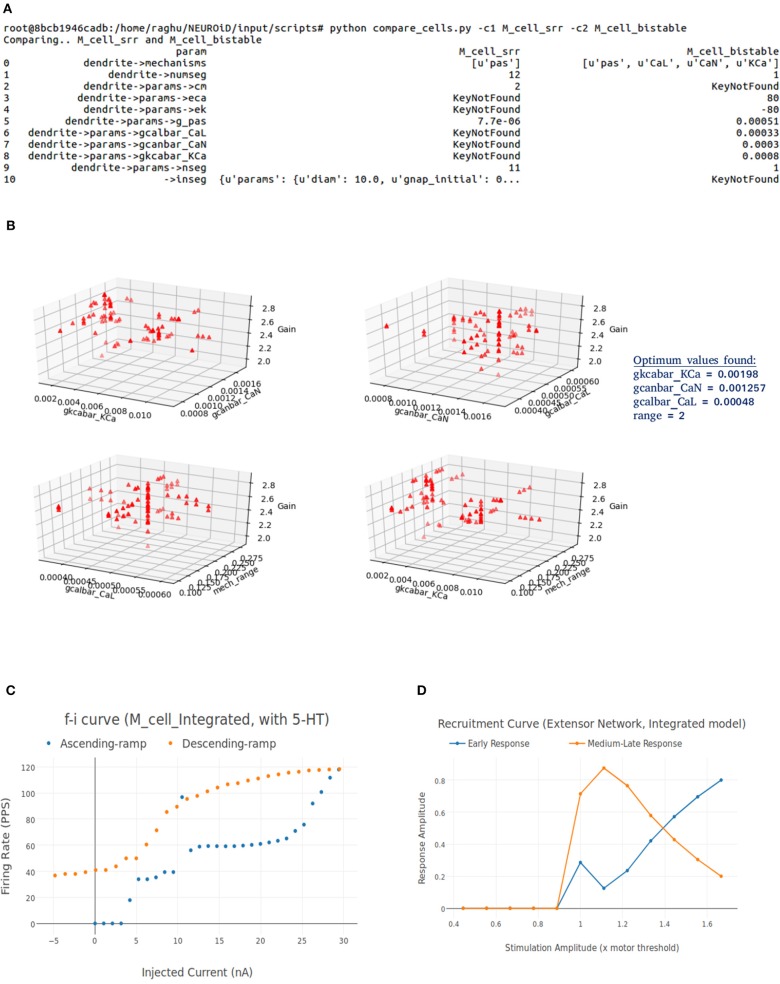
**(A)** The tabulated results of *compare*_*cell* tool, showing the difference in cell model parameters for *M*_*cell*_*srr* and *M*_*cell*_*bistable*. **(B)** Four parameters (gkcabar_KCa, gcanbar_CaN, gcalbar_CaL, range) were identified to be tuned using coordinate ascent search. For every set of parameters, we evaluate the integrated cell model by measuring the gain, which is a measure of closeness of the constituent model response (bistable firing property and spinal reflex recruitment curves) to the desired response. The parameters are plotted two at a time for visualization. **(C)** The bistable firing property of *M*_*cell*_*bistable* is also observed in the integrated model. **(D)** The spinal reflex recruitment curves elicited by EES were also observed with the integrated motoneuron cell model. The medium-late response reduces to less than 20% of its peak value when input stimulation is close to twice the motor threshold. These early and medium-late response in this curve is similar to the response observed with *M*_*cell*_*srr*.

Figure 13A shows the output of compare_cells tool which was used to compare the parameters of M_cell_srr and M_cell_bistable. The output of the script contains three columns. The first column shows the parameter that is being compared, while the other two columns show the parameter value in the two cell models being compared. If a parameter is not defined explicitly in a cell model template, the corresponding entry is shown as KeyNotFound. The initial values for all the parameters in our integrated model were chosen to match the values in M_cell_srr.

We identified gkcabar_KCa, gkcanbar_CaN, gkcalbar_CaL, and range (the indices of perisomatic sections where the CaL, CaN, and KCa mechanisms were inserted) as the parameters whose values need to be modified in the integrated model so that the model can display the properties of both the constituent models.

For every set of parameters, we evaluate the integrated cell model by measuring the constituent model properties (bistable firing property and spinal reflex recruitment curves). The direction and magnitude for change in each of the parameter is decided based on the closeness of the response of the current model with the desired system response.

Figure 13B shows the pairwise plots of the parameters that were searched during the coordinate ascent. Each triangle in the plot indicates one set of parameters that were evaluated. When we ran the coordinate ascent on a parameter space consisting of all the four parameters for 50 iterations, we found that the top-1 and top-2 (which was 98% of the top-1 result for the defined objective function) result gave us parameters that resulted in an integrated model that demonstrated all the constituent model responses such as bistable firing and spinal reflex recruitment curves.

In Figure 13C, we see that the integrated cell model displays the bistable firing property observed in M_cell_bistable. Then, we ran the experiment to obtain the spinal reflex recruitment curves on the new model definition. Figure 13D, shows the reflex recruitment curves obtained on the integrated cell model. The spinal recruitment curves of extensor network observed in Courtine et al. (2016) are shown in Figure 8C for comparison. We also plotted the FI curve and spike rate adaptation of the integrated cell model. The rheobase and steady state firing frequency was observed to match with that of the S type motoneuron values from Cisi and Kohn (2008).

An interesting observation made during the integration process was that it was necessary to add the CaL, CaN and KCa mechanisms in the perisomatic dendritic sections of the model. Adding them in all dendritic sections or in the distal dendritic sections resulted in models that could not reproduce results from one or the other of the constituent models. This observation concurs with recent findings (Manuel et al., 2014) that the L-type current is closer to the soma.

## 4. Discussion

In this paper, we have presented NEUROiD, a novel NEUROmotor integration and Design platform that allows systematic and structured integration of component models to create a neuromotor system. Using the human ankle and its controlling spinal circuitry as an example, we have demonstrated the use of NEUROiD in definition, simulation and visualization of the anatomy, physiology, and biomechanics at micro, meso and macro scales. This simultaneous visibility into multiple scales and discipline (Figure 10) is a contribution toward enabling construction of larger neuromotor system models. Further, enabling a concise definition of connection rules and repeating network motifs allow modelers to exploit recurring patterns for automatic or semi-automatic generation of neural circuits.

Various computational models reproduce one or the other aspect of the true biological system. In moving toward a model that shows increasing likeness to the true biological system, it may be important to create a single integrated model that demonstrates the system responses of each of the component models. It is also possible that the integrated model may be called upon to demonstrate an emergent property that was not apparent in any of the component models. In this work we demonstrated the broad contours of a method for the same. In the first step, tools provided within the NEUROiD platform were used to compare and merge models. This was followed by identification of the joint parameter space and iterative model tuning coordinate ascent/descent in the parameter space in order to converge toward a set of desired system responses. Though there have been efforts to explore creation of models without the need for any parameter tuning (Markram et al., 2015), it is resource intensive and involves building models from primary imaging data. However, when it is required to make use of the existing large database of models to create modular systems by integration, parameter tuning is inevitable.

Electrical stimulation modalities such as epidural electrical stimulation (EES) and trans spinal electrical stimulation (tsES) have shown promise as neuromodulation therapies for spinal cord injury and other motor deficits (Cogiamanian et al., 2012; Courtine et al., 2016). Designing electrical stimulation protocols are non-trivial as the designer is required to predict the effect of anatomical placement, orientation of stimulating electrodes, and current waveform characteristics. Frequency and intensity of stimulation is known to affect the recruitment patterns of neuronal cells (Courtine et al., 2016). Stimulus received by spinal cord elements are intricately related to cell sizes, orientations, and positions with respect to the electrodes, anatomy, cellular and synaptic electrophysiology and morphology of spinal cord (Kuck et al., 2017, 2018).

Anatomically accurate models are necessary to understand the effects of therapeutic stimulation on neuromuscular action. The multiscale nature of NEUROiD allows creation of models of neuromotor systems under consideration at the right scale for the question being asked. For instance, if the objective is to understand the differential effect of a stimulation protocol on cells with different orientations, then models must factor in the cell morphology and orientation. In the context of EES, it might be more important to model the anatomy of the dorsal roots. The electric field setup in the spinal cord due to a given stimulation configuration can be generated using tools like COMSOL or built into NEUROiD itself. Models of injured or severed spinal cords can also be used in understanding pathology and regeneration in spinal cord injury.

Emerging technology areas often require simulation of human nervous and muscular systems for a wide variety of external stimuli. For example, game programming (Sanchez-Crespo and Dalmau, 2004) and popular gaming engines^8^ are typically physics based. Computational models of neuromuscular functions (Valero-Cuevas et al., 2009) could be used to derive biophysically realistic responses of gaming characters and make them more realistic. There is also a greater push toward *in silico* simulation in the biomedical industry (Viceconti et al., 2016). A multi-scale system simulation tool will play an important role during the design, development, and testing of drugs. Development of medical devices and prosthetics will also be accelerated if their interactions could be tested on a multi-scale simulation platform before clinical trials. These industry trends are still in their infancy and will probably take a long time to materialize. However, there is certainly a need for platforms to accelerate *in silico* design and development.

The client-server architecture of NEUROiD allows the compute intensive operations to be performed on a high-performance server. We are cognizant of the challenges involved in simulating large biologically realistic networks and intend to employ suitable CPU/GPU parallelization techniques, such as (Ben-Shalom et al., 2013; Hoang et al., 2013; Vooturi et al., 2017) in future releases of NEUROiD to improve the simulation time. Integration of other parameter search techniques such as Van Geit et al. (2016) and Sutton et al. (1999) lie within the scope of future work to help the users in efficient curation of models. With continuous integration of multiple neuromotor models, we hope that NEUROiD can simulate multiple neuromotor and movement behaviors in the future.

## Author Contributions

MR provided the oversight and intellectual direction for the project, reviewed experimental results, manuscript, and source code. RI implemented the bulk of the source code for NEUROiD, built the models described in this paper on NEUROiD, built and integrated various opensource models, validated the results, and created the manuscript. MP implemented parts of NEUROiD source code, built a configurable and automatic motor circuits generation tool and integrated it with NEUROiD, explored the compatibility of OpenSim and other opensource models with NEUROiD, performed extensive literature survey, curated the spinal cord information, and drafted them into NEUROiD. AS was instrumental in helping us integrate LFPSim and other opensource models into NEUROiD.

### Conflict of Interest Statement

The authors declare that the research was conducted in the absence of any commercial or financial relationships that could be construed as a potential conflict of interest.

## Footnotes

^1^https://www.humanbrainproject.eu/en/brain-simulation/brain-simulation-platform/

^2^https://www.json.org

^3^https://plot.ly/javascript/

^4^https://nodejs.org/

^5^https://www.mozilla.org/en-US/firefox/new/

^6^https://threejs.org

^7^https://www.neuron.yale.edu/neuron/static/new_doc/programming/hocsyntax.html

^8^https://unity3d.com/
