# Supplementary material for: Curated Model Development Using NEUROiD: A Web-Based NEUROmotor Integration and Design Platform
Source: Front Neuroinform. 2019 Aug 7;13:56. doi: 10.3389/fninf.2019.00056 (PMC6693358; doi:10.3389/fninf.2019.00056)
Supplement: Supplementary file 1 [file Table_1.DOCX]

Supplementary Material

# NEUROiD

NEUROiD stands for NEUROmotor integration and Design platform. It is designed to have an input interface that even non-programmers are comfortable with (e.g., xls). Another important design goal of NEUROiD was to have all the simulations be performed on a different machine (possibly remote) and connect to the client (user) using standard protocols (http/https). This enables the development and improvement of models at one place which can then be utilized by many users.

For all the sections in this paper, we assume that NEUROiD is run as a server on the same machine as the client.

NOTE: A copy of paper, supplementary material, videos and install script is available at:

<https://drive.google.com/drive/folders/1fl4RvXM_ICuGaY-tRRYUqdt6DjfMtoUj?usp=sharing>

# Getting familiar with NEUROiD interface

In this section, we describe the NEUROiD interfaces, features and typical usage examples. Here, we assume that the prebuilt ubuntu docker with NEUROiD is already setup.

## Installing NEUROiD docker

Run the neuroid_release.py script provided. This will setup NEUROiD environment in a docker.

NOTE: NEUROiD is currently supported on Linux only (tested on Ubuntu 16.04 and centos 7)

*python neuroid_release.py -a ci -t paper_v1.1*

*python neuroid_release.py -a ls -t paper_v1.1*

## Setting up NEUROiD for an experiment

Setting up NEUROiD for an experiment involves three steps:

1. Parse user input and create platform agnostic spine.json

*cd /home/raghu/NEUROiD/*

*./run.sh*

These commands invoke a series of scripts to parse the user input files and create a platform agnostic model of neuromotor system. The spine.json file is created in /home/raghu/NEUROiD/ folder on successful completion.

By default, this initializes L4 and L5 segments of spinal cord.

1. Start the node server

*cd /home/raghu/NEUROiD/server_client/server/node_modules*

*nodejs server.js is*

These commands start the node server

The log “NEUROiD *listening on port 3000!*” will be seen on the terminal on successful completion of the command

1. Connect to the server from client and run experiments

*cd /home/raghu/bin/firefox/*

*./firefox*

These commands start a firefox browser instance. Note that the docker container has firefox browser of version 57.0b9.

Node server can be accessed by using the url “*http://localhost:3000/index.html?load=demo/spine.js”* on the browser

A screen similar to the one shown below will be rendered on successful connection to the node server


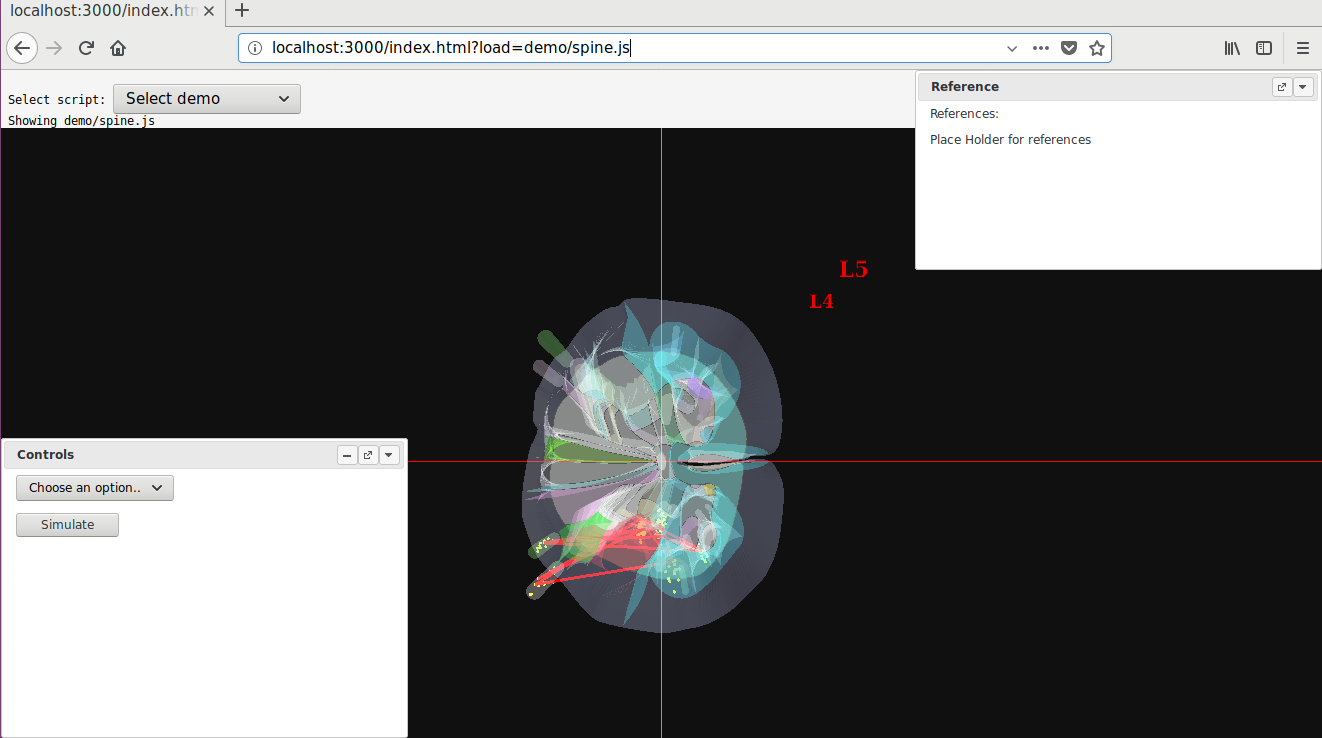


Figure 1: Initial NEUROiD Screen

NOTE:

NEUROiD is not (yet) compatible with latest versions of firefox. Firefox updates automatically. If you find that the screen above is not rendered, please check the version of firefox. If it is not 57.0b9, then:

- Delete the directory /home/raghu/bin/firefox

- Extract firefox again: cd /home/raghu/bin; tar -xf firefox-57.0b9.tar.bz

Note that the above steps uses the default values from user input xls files to setup NEUROiD. This is sufficient to run the experiments listed in the paper. The user can modify any of the parameters in the xls files and re-run the setup procedure to run experiments with modified parameters.

## Performing experiments with NEUROiD

Click on the “choose an option” and select “Save/Restore setup” from the control panel shown on the bottom left part of the NEUROiD view


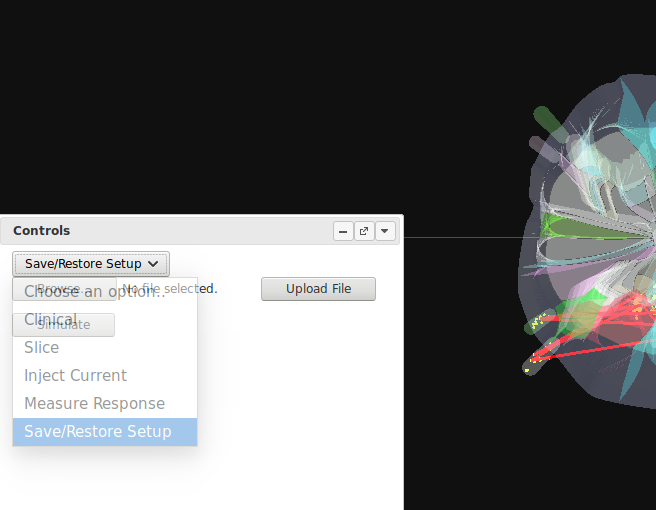


Figure 2: “Save/Restore” option to load an experimental setup json file

Then select one of the setup json files present in the /home/raghu/NEUROiD/input/demos folder


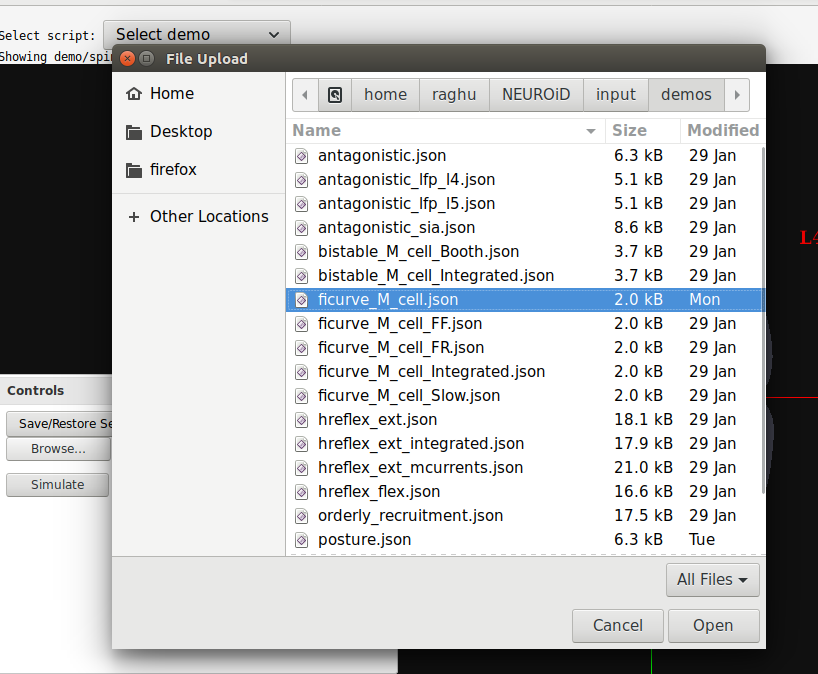


Figure 3: Select and load the experiment setup json file

Click on upload button. This will setup any input injections (red) and measurement (blue) probes necessary for the experiment.


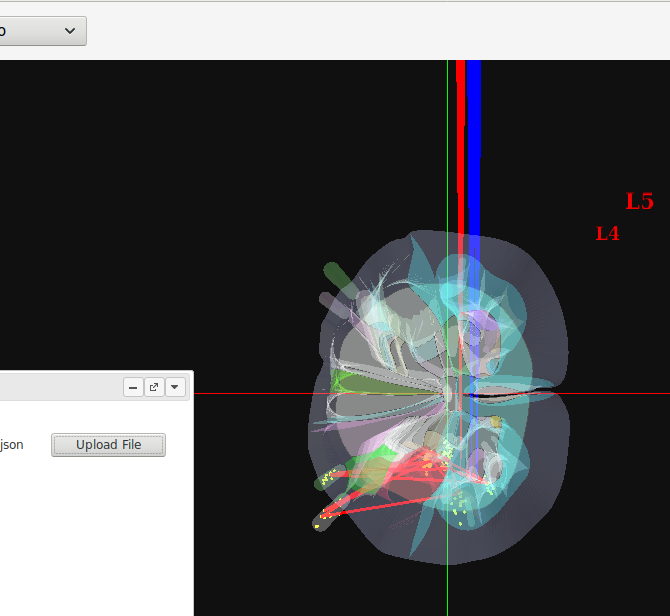


Figure 4: Display of markers (injection and measurement) based on experimental setup

Click “simulate” to perform the experiment and display the results. The definition of experiment to be run, input to be given, measurements to be done and displayed are all defined in the setup json file. Users can modify the json file to change the parameters of experiment and re-run the experiments.

Below are some sample plots from the experiment whose setup is defined in the /home/raghu/NEUROiD/input/demos/ficurve_M_cell.json


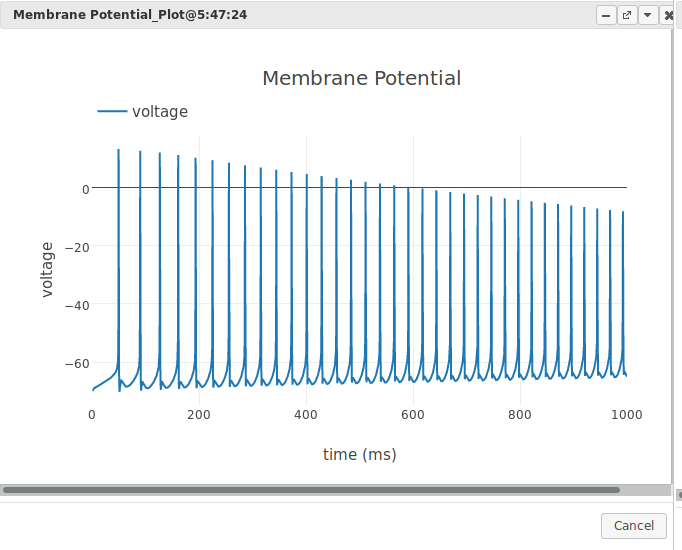


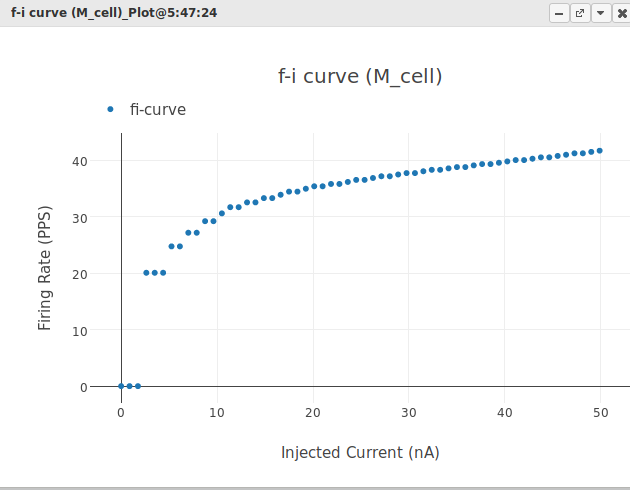


Figure 5: Results of experiment are displayed in dialog boxes on the browser

# Peek inside NEUROiD

Here, we provide a brief overview of the source file organization of NEUROiD. Please refer to the documents and comments in source for further information

Below figure shows the directory tree structure of NEUROiD source code

*root@b6ed7b3d0746:/home/raghu/NEUROiD# tree -L 2*

*.*

*├── extensions*

*│   ├── m_cell_reference*

*│   └── par_search*

*├── input*

*│   ├── README.md*

*│   ├── README_model_Define.xlsx*

*│   ├── README_simulation_demos.pdf*

*│   ├── model*

*│   ├── scripts*

*│   └── simulation*

*├── model*

*│   ├── README.md*

*│   ├── gen*

*│   └── run.py*

*├── output*

*├── run.sh*

*├── server_client*

*│   ├── README.md*

*│   ├── client*

*│   ├── server*

*│   └── test-suite*

*└── settings.ini*

*14 directories, 8 files*

NEUROiD workflow can be classified into three major steps:

1. Model definition and generation
2. Client-server interactions
3. Simulation and display of results

Model definition and generation:

$NEUROiD_HOME/input/model contains the model definitions. The directory tree structure of this folder is given below

*root@b6ed7b3d0746:/home/raghu/NEUROiD/input/model# tree -L 3*

*.*

*├── Define*

*│   ├── Cellular*

*│   │   ├── Anatomy*

*│   │   ├── Biomechanics*

*│   │   └── Physiology*

*│   ├── Network*

*│   │   ├── Anatomy*

*│   │   ├── Biomechanics*

*│   │   └── Physiology*

*│   └── Systems*

*│   ├── Anatomy*

*│   └── Biomechanics*

*└── Visualize*

*└── settings.xlsx -> ../.files/xls/settings.xlsx*

*13 directories, 1 file*

A. The $NEUROiD_HOME/input/model/Define/Network/Anatomy/contours_images contains the manually pre-processed scanned images of spinal sections.

These images are used by the script $NEUROiD_HOME/model/gen/image_to_contours.py to generate the contours for each lamina in the image.

The obtained contours are manually mapped to the regions defined in atlas and the contours are stored in $NEUROiD_HOME/input/model/Define/Network/Anatomy/contours to be used for simulation

B. All the xlsx files in the $NEUROiD_HOME/input/model folder can be modified by the user to change the model definition parameters. The xlsx files are organized into [micro|meso|macro]/[Anatomy|Physiology/Biomechanics] sub-folders representing the corresponding parameters in the 3x3x3 cublets.

Please refer to the README*.* file under $NEUROiD_HOME/input/ for more information on the files under the input directory.

Scripts responsible for model generation are present in the $NEUROiD_HOME/model folder. The directory structure of this folder is shown below

*root@b6ed7b3d0746:/home/raghu/NEUROiD/model# tree -L 2*

*.*

*├── README.md*

*├── gen*

*│   ├── continuous_regions_display.py*

*│   ├── create_cell_templates.py*

*│   ├── create_model.py*

*│   ├── create_template_map.py*

*│   ├── gen_new_contour.py*

*│   ├── image_to_contours.py*

*│   ├── model_utils.py*

*│   ├── motor_ckts.py*

*│   ├── place_cells.py*

*│   ├── process_contours.py*

*│   └── utils.py*

*└── run.py*

*1 directory, 13 files*

$NEUROiD_HOME/model/run.py is the main script to generate the model. This invokes the functions implemented in other scripts defined under $NEUROiD_HOME/model/gen. On successful execution, this script generates the $NEUROiD_HOME/output/spine.json that contains the model definition.

Please refer to the $NEUROiD_HOME/model/README.md file for more information on model definition and scripts used to obtain the model definition.

Client-Server interactions

$NEUROiD_HOME/server_client contains all the source for client and server interactions

*root@b6ed7b3d0746:/home/raghu/NEUROiD/server_client# tree -L 2*

*.*

*├── README.md*

*├── client*

*│   ├── data -> /home/raghu/NEUROiD/model/../output*

*│   ├── image.jpg*

*│   ├── index.html*

*│   ├── lib*

*│   └── model*

*├── server*

*│   ├── node_modules*

*│   └── simulate*

*└── test-suite*

*├── README.md*

*├── plot_py.py*

*├── results*

*├── run_one_test.js*

*├── run_test.py*

*├── testcase.py*

*├── testcases*

*└── utils.py*

*10 directories, 9 files*

A. After creating the model definition json file, a nodejs server has to be started which will fetch the model definitions from the server and display it on the client.

Below commands start the nodejs server

cd $NEUROiD_HOME/server_client/server/node_modules

nodejs server.js

If the server starts successfully, the below message is printed on console:

"NEUROiD listening on port 3000!"

B. After the server is started successfully, client (browser) can connect to the server and fetch the model definitions necessary to display the model

Below commands start a browser:

cd /home/raghu/bin/firefox; ./firefox

$NEUROiD_HOME/server_client/client has all the client side scripts (javascripts) to handle user interactions and visualizations.

C. $NEUROiD_HOME/server_client/server/simulate contains all the python scripts that are responsible to run NEURON simulation, store and format the results of experiments

Please refer to the $NEUROiD_HOME/server_client/README.md file for more information on the organization of client and server in NEUROiD

# Setup procedure and json files for experiments described in paper

To perform any of the experiments listed here, perform the setup procedure described here, load the setup json file (as described in section 2.3) and run the simulation.

| **ID** | **Experiment** | **Description** | **Setup** | **Setup json file name** |
| --- | --- | --- | --- | --- |
| 1 | Fi curve (M cell) | Frequency Vs injected current plot for Motoneuron | Basic^1^ | ficurve_M_cell.json |
| 2 | Fi curve (FF cell) | Frequency Vs injected current plot for fast fatigue type Motoneuron | Basic^1^ | ficurve_M_cell_FF.json |
| 3 | Fi curve (FR cell) | Frequency Vs injected current plot for fatigue Resistant type Motoneuron | Basic^1^ | ficurve_M_cell_FR.json |
| 4 | Fi curve (Integrated cell) | Frequency Vs injected current plot for Integrated Motoneuron | Basic^1^ | ficurve_M_cell_Integrated.json |
| 5 | SRA curve (M cell) | Spike Rate Adaptation curve for Motoneuron | Basic^1^ | sracurve_M_cell.json |
| 6 | SRA curve (Integrated cell) | Spike Rate Adaptation curve for Integrated Motoneuron | Basic^1^ | sracurve_M_cell_Integrated.json |
| 7 | Bistable property (M cell) | Bistable property of Motoneuron as described by [Booth 1999] | Basic^1^ | bistable_M_cell_Booth.json |
| 8 | Bistable property (Integrated cell) | Bistable property of Integrated Motoneuron obtained by | Basic^1^ | bistable_M_cell_Integrated.json |
| 9 | Antagonistic response | Antagonistic responses of flexor-extensor controlling a simple ankle model | Basic^1^ | antagonistic.json |
| 10 | Antagonistic response with LFP | Antagonistic responses of flexor-extensor controlling a simple ankle model with measurements from an LFP probe placed in L4 segment | Basic^1^ | antagonistic_lfp_l4.json |
| 11 | Antagonistic response with OpenSim | Antagonistic responses of flexor-extensor controlling an ankle model in OpenSim | OpenSim^2^+Basic^1^ | antagonistic_sia.json |
| 12 | Reflex Recruitment curves for extensor (M cell) | Spinal Reflex Recruitment curves for extensor | Basic^1^ | srr_ext.json |
| 13 | Reflex Recruitment curves for flexor (M cell) | Spinal Reflex Recruitment curves for flexor | Basic^1^ | srr_flex.json |
| 14 | Reflex Recruitment curves for flexor (Integrated cell) | Spinal Reflex Recruitment curves for flexor with the Integrated motoneuron cell model | IntModel^3^+Basic^1^ | srr_ext_Integrated.json |
| 15 | Orderly Recruitment | Demonstrates the orderly recruitment of Slow, FF and FR type motoneurons | Orderly^4^+Basic^1^ | orderly_recruitment.json |

^1^ The setup is default setup of NEUROiD. Refer to section 2.2 for instructions

^2^ The OpenSim application running the ankle model can be started using below commands:

*cd /home/raghu/NEUROiD/output/opensim*

*python neuroid_osim_glue.py*

^3^ The changes to replace the default Motoneuron cell model with Integrated cell model are as below:

*cd /home/raghu/NEUROiD/input/.files/xls*

*cp srr_integrated/* .*

*This command copies cell_template_xlsx file that has below changes:*

*Replace all instances of M_cell with M_cell_Integrated (2 instances in default file, i,e, cells C2 and C7*

^4^ The changes to add Slow, FR and FF type motoneuron cell groups is as given below:

*cd /home/raghu/NEUROiD/input/.files/xls*

*cp orderly_recruitment/* .*

*This command copies cell_template_xlsx and other xlsx file that have changes to add the Slow, FR and FF Cellgroups into the model*

# Structure of json files

Below diagram shows the main fields in the *model definition* json file.

#
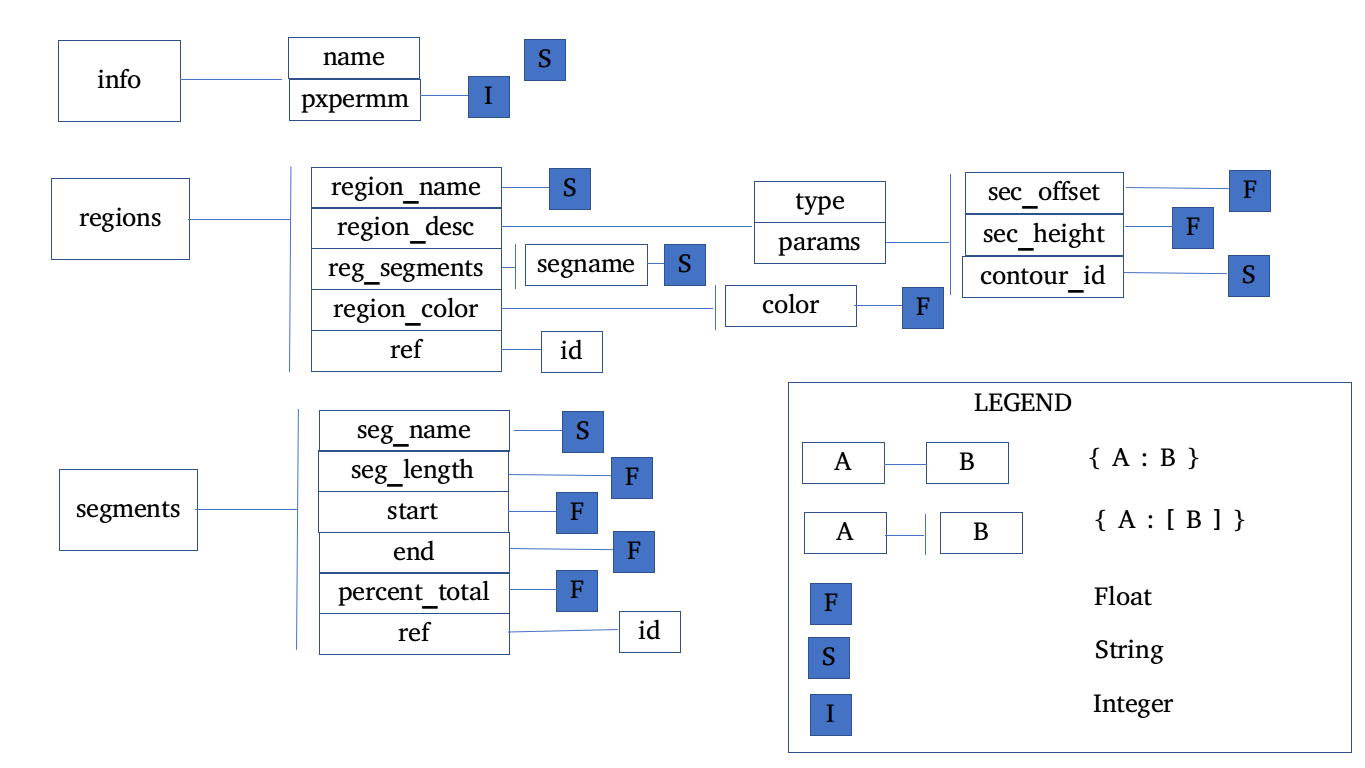


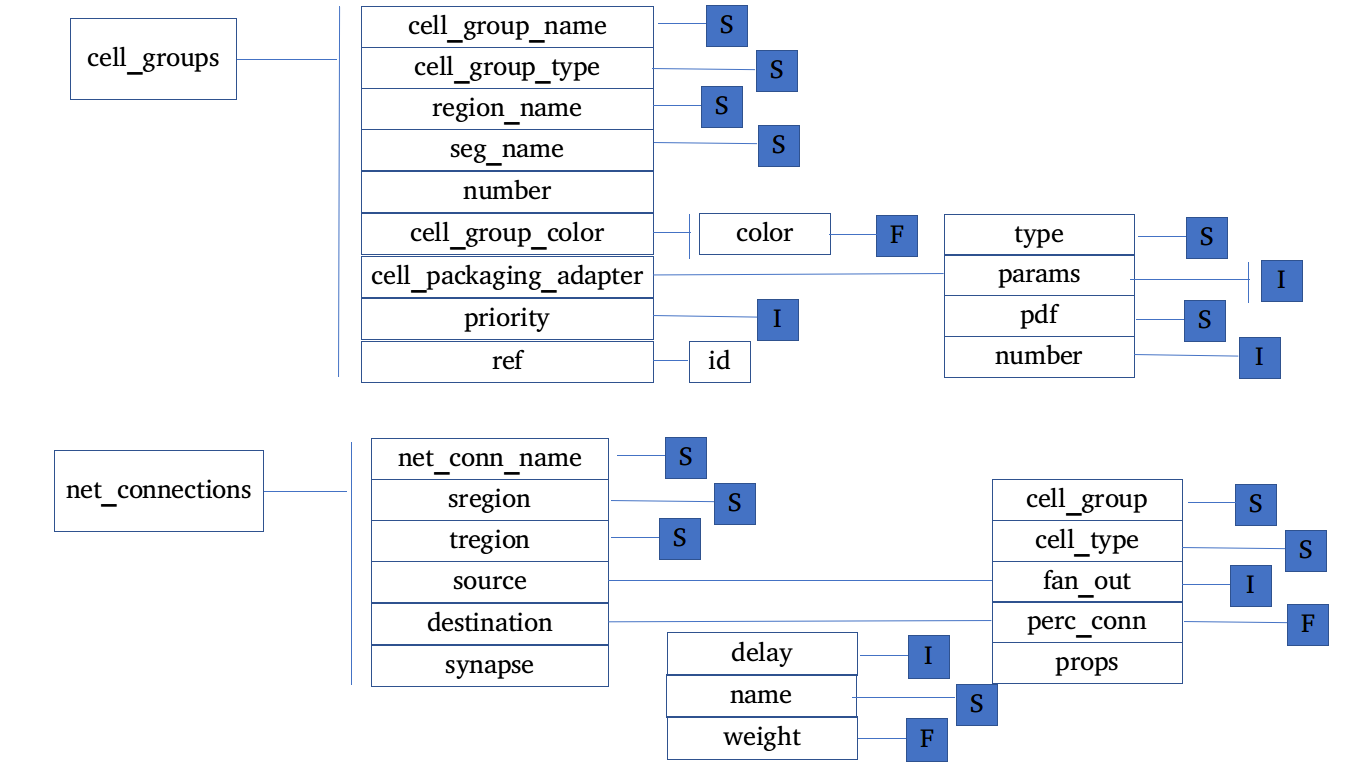


Below diagram shows the main fields in the json file containing *simulation definition*


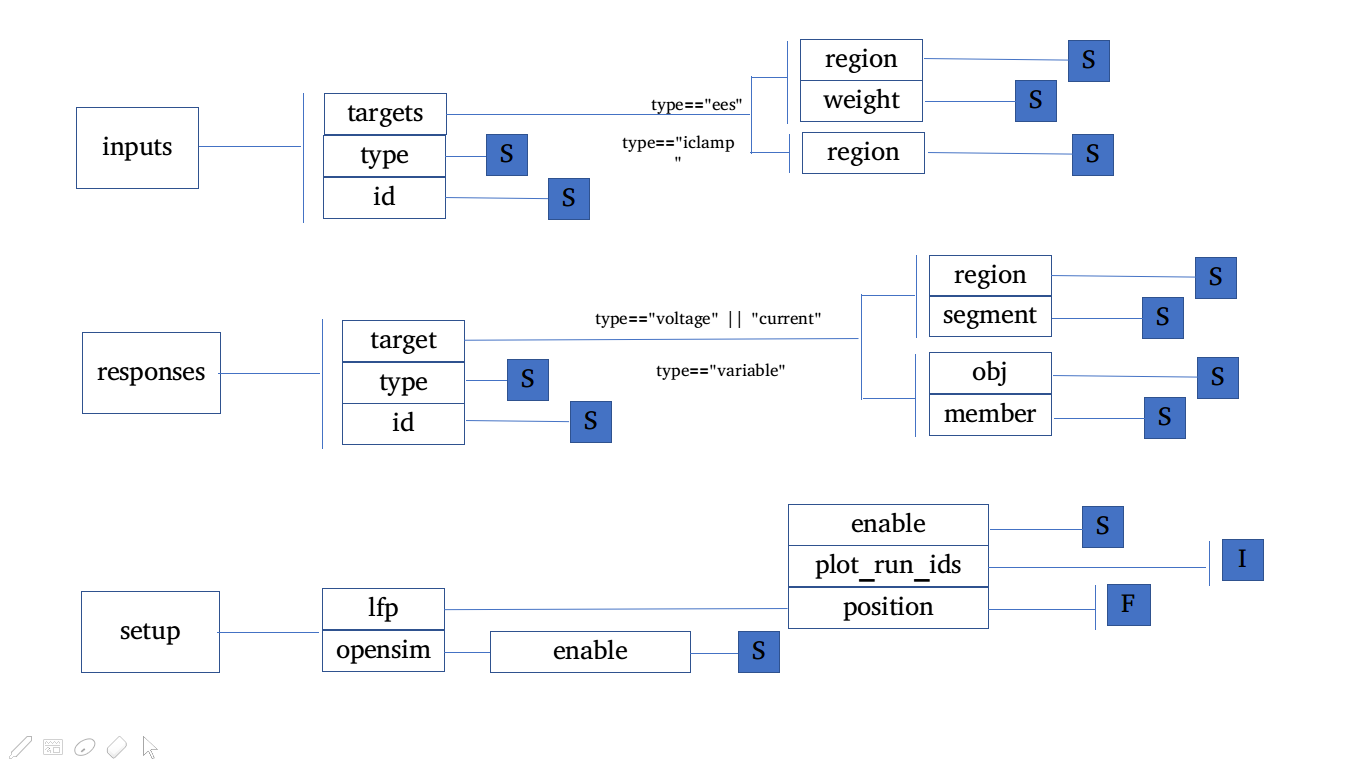


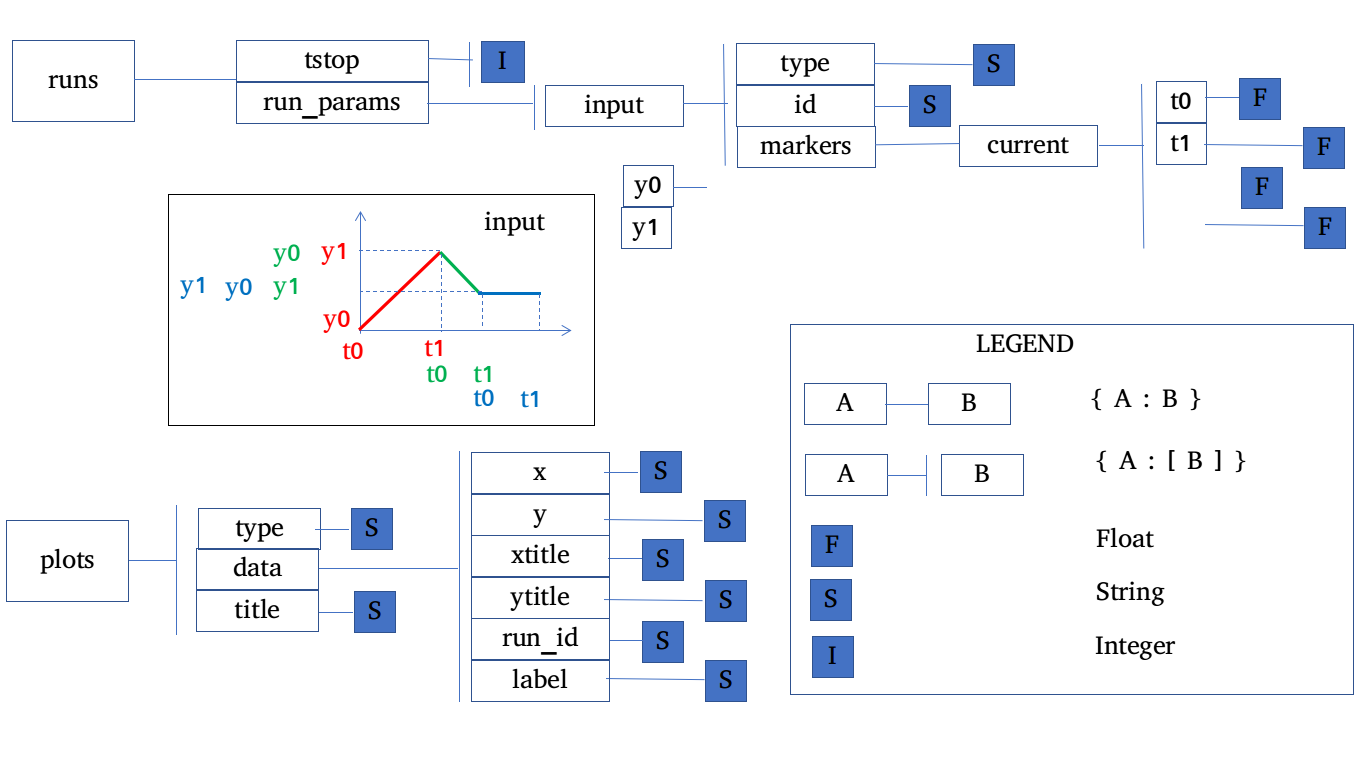


# Further details

*NEUROiD has other README files along with source code providing more details into individual components. Please refer to these for more information. The list of README files is given below:*

*$NEUROiD_HOME/input/README.md*

*$NEUROiD_HOME/input/README_simulation_demos.pdf*

*$NEUROiD_HOME/input/README_model_Define.xlsx*

*$NEUROiD_HOME/input/README_TemplateConnections.docx*

*$NEUROiD_HOME/server_client/README.md*

*$NEUROiD_HOME/server_client/test-suite/README.md*

*$NEUROiD_HOME/model/README.md*
